# The signaling pathways in obesity‐related complications

**DOI:** 10.1002/ccs3.12039

**Published:** 2024-06-07

**Authors:** Preethi Chandrasekaran, Ralf Weiskirchen

**Affiliations:** ^1^ UT Southwestern Medical Center Dallas Dallas Texas USA; ^2^ Institute of Molecular Pathobiochemistry Experimental Gene Therapy and Clinical Chemistry (IFMPEGKC) RWTH University Hospital Aachen Aachen Germany

**Keywords:** cardiovascular disorders, dyslipidemia, hypertension, MAFLD/MASLD, obstructive sleep apnea, polycystic ovary syndrome, type 2 diabetes mellitus

## Abstract

Obesity, a rapidly expanding epidemic worldwide, is known to exacerbate many medical conditions, making it a significant factor in multiple diseases and their associated complications. This threatening epidemic is linked to various harmful conditions such as type 2 diabetes mellitus, hypertension, metabolic dysfunction‐associated steatotic liver disease, polycystic ovary syndrome, cardiovascular diseases (CVDs), dyslipidemia, and cancer. The rise in urbanization and sedentary lifestyles creates an environment that fosters obesity, leading to both psychosocial and medical complications. To identify individuals at risk and ensure timely treatment, it is crucial to have a better understanding of the pathophysiology of obesity and its comorbidities. This comprehensive review highlights the relationship between obesity and obesity‐associated complications, including type 2 diabetes, hypertension, (CVDs), dyslipidemia, polycystic ovary syndrome, metabolic dysfunction‐associated steatotic liver disease, gastrointestinal complications, and obstructive sleep apnea. It also explores the potential mechanisms underlying these associations. A thorough analysis of the interplay between obesity and its associated complications is vital in developing effective therapeutic strategies to combat the exponential increase in global obesity rates and mitigate the deadly consequences of this polygenic condition.

## Graphical abstract

Obesity is associated with a variety of long‐term complications that result from being overweight.

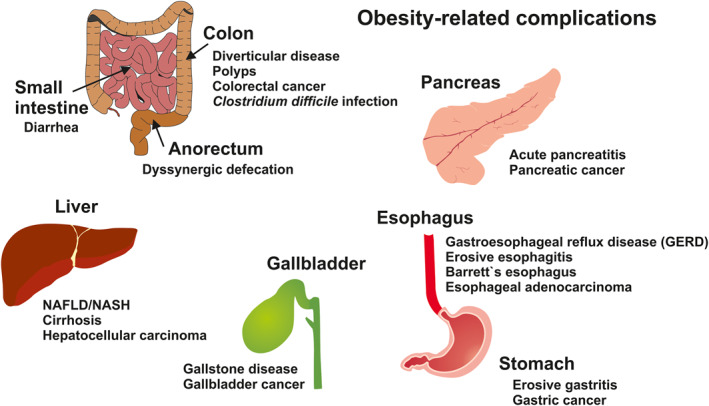

## 1 INTRODUCTION

It is estimated that approximately 1 billion adults will become obese, and 177 million adults are at risk of becoming severely obese by 2025 worldwide.^1^ Additionally, obesity is associated with increased mortality in numerous disorders, with nearly 1 in 5 deaths attributed to obesity.^2^ It is alarming to note that adults aged 35–59 years with morbid obesity, defined as body mass index (BMI) greater than or equal to 40 kg/m^2^, are 5 times more likely to die from heart failure and cardiovascular diseases (CVDs), 6.5 times at risk of dying from stroke, and, more strikingly, 22.5 times at risk of dying from complications associated with diabetes mellitus (DM), compared to the adult population with a normal BMI.^2^ In fact, obesity is identified as an independent risk factor of CVDs and their associated mortality. Recently, waist circumference and waist‐hip ratio have been widely used to predict cardio‐metabolic complications as they are better measures of abdominal obesity.^3^ Although BMI is the simplest and most widely used measure to assess obesity, it does not differentiate between lean muscle and fat mass and does not predict the distribution of body fat. Consequently, waist circumference and waist‐hip ratio are supposed to be better predictors of obesity related risks compared to BMI.^4^ In 2014, adult obesity affected around 10.8% of men and 14.9% of women globally. If this trend continues, it is estimated that by 2025, 18% of men and 21% of women will become obese worldwide.^5^

An interesting meta‐analysis of 239 prospective studies, involving 10.6 million individuals from various regions including North America, Asia, and Australia, revealed that the population with a BMI range of 20–25 kg/m^2^ had the lowest all‐cause mortality rates. In contrast, overweight and obese populations experienced a significant increase in mortality.^6^

The increase in adiposity and its connection to numerous complications can be attributed to both structural and metabolic effects. Subcutaneous adipose tissue primarily functions to store excess calories as triglycerides through adipocyte hypertrophy, which in turn protects vital organs like the liver, heart, and kidneys. However, if the capacity of subcutaneous adipose tissue exceeds a certain threshold, hypertrophied adipocytes can rupture and trigger inflammation, leading to the deposition of triglycerides within visceral adipose tissue.^7^ The metabolic effects associated with obesity‐related complications are primarily caused by the stimulation of pro‐inflammatory cytokines (such as TNF‐α, IL‐1, IL‐6) and lipotoxicity resulting from increased levels of free fatty acids (FFAs) and lipid intermediates like ceramides, which are implicated in insulin resistance, DM, MASLD and CVDs.^8^

Multiple signaling pathways are involved in the complex pathophysiology of obesity and its related complications. The PI3K/AKT pathway, closely related to insulin signaling is involved in the regulation of various physiological processes and is crucial for maintaining metabolic homeostasis.^9^ Its function in insulin‐sensitive tissues is interconnected with obesity and obesity‐related complications. Moreover, adipose tissue inflammation caused by the activation of mitogen‐activated protein kinases (MAPKs) plays a key role in adipogenesis. Additionally, MAPKs cause glucose intolerance in obese states by directly inactivating IRS1 and indirectly inactivating PPARγ. Furthermore, the JAK‐STAT pathway participates in leptin ‐mediated anorectic effects and regulates fat accumulation in the liver. Finally, other signaling mechanisms include the AMPK pathway, whose activation causes weight gain, TGF‐β signaling involved in the regulation of glucose homeostasis and endoplasmic reticulum (ER) signaling pathways in which the accumulation of unfolded protein response in the ER causes metabolic dysfunction.^10^

This narrative review aims to summarize the major pathophysiological connections between obesity and its complications, by analyzing the pathways and key features of the signaling mechanisms that underlie this association.

## 2 SIGNALING PATHWAYS INVOLVED IN THE PATHOPHYSIOLOGY OF OBESITY‐ASSOCIATED COMPLICATIONS

**FIGURE 1:**
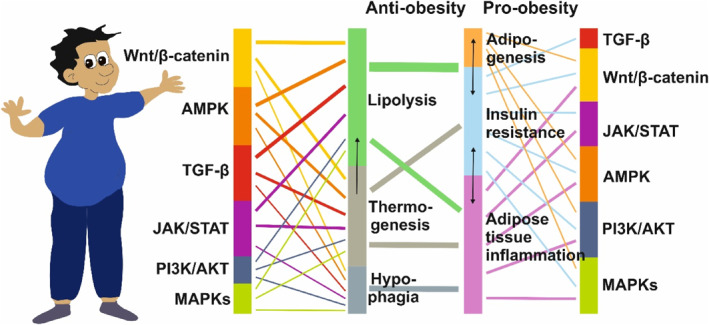
The complexity and interconnected nature of crucial signaling pathways that mediate signals either promoting or counteracting obesity. There are numerous pathways involved in promoting or counteracting obesity, which impact appetite, thermogenesis, lipolysis, adipose tissue metabolism, glucose and fat homeostasis, adipogenesis, and energy expenditure. Key pathways include MAPKs, PI3K/AKT, JAK/STAT, TGF‐β, AMPK, and Wnt/β‐catenin. These pathways are interconnected and can have both stimulating and inhibiting effects. For example, the AMPK pathway directly affects lipolysis while also promoting insulin resistance and inflammation in adipose tissue. Another example is the JAK/STAT pathway, which has anti‐obesity effects by impacting thermogenesis, lipolysis, and hypophagia, while also promoting the pathogenesis of obesity by impacting adipose tissue inflammation and insulin resistance. The various activities and interconnection of the pathways depicted make it highly difficult to develop suitable drugs. This figure has been redrawn in a modified form from the work of Wen and colleagues.^10^

An in‐depth knowledge of the signaling pathways involved in obesity‐associated complications is crucial for gaining a better understanding of the mechanistic insights needed for targeted therapeutic approaches. However, there are direct and indirect effects that impact an individual's susceptibility to obesity, which results from excessive calorie consumption and/or insufficient calorie expenditure. Unfortunately, the underlying metabolic processes are influenced by numerous signaling pathways that can either promote or counteract obesity (Figure 1).

In the following, we will discuss some of the important signaling cascades.

### 2.1 Mitogen‐activated protein kinase pathways

Several studies have shown that the three tiered cascade of (MAPKs) is essential for signal transduction. The MAPKs ERK1/2, c‐Jun N‐terminal kinase (Jun N‐terminal kinase (JNK)) and p38, play crucial roles in regulating adipogenesis, glucose homeostasis, and thermogenesis.^11^ Specifically, the activation of MAPK stimulates adipose inflammation in obesity. This was demonstrated in a study showing that inhibiting the caspase recruitment domain 9/MAPK pathway led to reduced inflammation, improved glucose tolerance, and decreased adipocyte enlargement.^12^ CARD9 is an adapter protein associated with immune cell activation and inflammatory responses. Previous studies have shown that CARD9 overexpression activates the kinases p38 and JNK, which are essential for producing proinflammatory cytokines.^13^ The study by Zeng et al. further found that Card9^−/−^ mice had lower mRNA expression of p38 MAPK, JNK and ERK compared to wild‐type mice.^12^ Another study used multiomics analysis in mice fed a high‐fat diet and showed that inflammatory genes are enriched in the ERK MAPK pathway, particularly in macrophages.^14^ MAPKs also plays a vital role in causing insulin resistance by directly inactivating insulin receptor (INSR) substrate (IRS)‐1 and indirectly activating PPAR‐γ.^15^, ^16^ The phosphorylation of PPARγ by ERK enhances the ability of transcriptional coactivator with PD2‐binding motif to negatively regulate PPARγ, causing impaired insulin sensitivity. The p38 MAPK pathway maintains glucose homeostasis in obesity by enhancing mRNA stability and nuclear migration of X‐box binding protein in the liver.^17^ Under obese conditions, chronic inflammation leads to insulin resistance in adipose tissue. p38α functions as a central mediator of *β*‐adrenergic induced uncoupling protein 1 expression in brown adipocytes. In white adipocytes, inactivation of p38α leads to increased reprogramming from white to beige adipocytes and resistance to diet‐induced obesity.^18^, ^19^ p38α controls lipolysis and protects against fatty liver in hepatocytes.^20^ This was evidenced by the development of severe steatohepatitis in high fat diet fed liver specific p38α knockout mice.^20^ Licochalcone F, a synthetic retrochalcone, was found to inhibit TNF‐α‐induced expression of inflammatory factors, reduce adipocyte size, decrease macrophage infiltration in white adipose tissue, and alleviate glucose intolerance by interacting with MAPK signaling pathways.^21^ It has been shown that Licochalcone F enhances AKT signaling and reduces p38 MAPK signaling in white adipose tissue. The anti‐inflammatory effects of Licochalcone F in alleviating obesity‐induced chronic inflammation are partially attributed to its ability to downregulate the p38 signaling pathway. In the central nervous system, ERK1/2 regulates appetite by enhancing glucose‐stimulated proopiomelanocortin (POMC) gene expression in hypothalamic neurons.^22^ Additionally, it has been demonstrated that leptin is modulated by JNK3 in high‐fat diet fed mice, specifically on Agrp neurons.^11^

Furthermore, MAPKs play crucial roles in the complex interaction between obesity and insulin resistance. The dual‐specificity phosphatase 9 (DUSP9), a cytoplasmic phosphatase, is known to dephosphorylate ERK1/2, JNK and ASK1 thereby controlling various MAPK pathway cascades. The dephosphorylation of multiple MAPKs such as ERK1/2, JNK, p38 and ASK1 kinases by DUSP9, restores the tyrosine phosphorylation of IRS1 alleviating glucose intolerance.^23^ Interestingly, JNK1 and JNK3 stimulate the serine/threonine phosphorylation of IRS1 and IRS2, which in turn induces insulin resistance.^24^ In brown adipose tissue (BAT), IL‐27, as well as the p38 MAPK/PGC‐1α signaling pathway, play a fundamental role in promoting adipocyte thermogenesis and energy expenditure.^14^, ^25^

### 2.2 The interconnection between JAK/STAT signaling pathway and obesity

The JAK/STAT signaling pathway is a crucial signal transduction pathway comprised of JAK1, JAK2, and JAK3, along with STAT1, STAT2, STAT3, STAT4, STAT5a, STAT5b, and STAT6. This pathway plays a direct role in obesity and interacts with MAPK or PI3K.^26^

The binding of cytokines and growth factors to their corresponding receptors leads to receptor dimerization and recruitment of related JAKs. Subsequently, the ligand‐receptor connection induces transphosphorylation of JAK. The activated JAK causes receptor tyrosine phosphorylation, forming a docking site for STATs. JAK phosphorylates STAT at the docking site, leading to the dissociation of STAT from the receptor to form heterodimers or homodimers through SH2‐domain phosphotyrosine interactions. The translocation of these dimers targets the gene promoters and regulates the transcription of target genes. STAT either binds to its DNA target site or forms a complex with non‐STAT transcription factors to regulate transcription. The non‐classical JAK/STAT signaling involves unphosphorylated STAT3 inducing multiple STAT3 target gene expressions without Ser727 phosphorylation and Lys685 acetylation.^27^

The positive regulators of JAK/STAT signaling include the formation of a glucocorticoid receptor complex with activated STAT5. This complex promotes STAT5‐dependent transcription. Additionally, the transcriptional coactivator CBP/p300 and p30 acts as auxiliary activators of STAT1α. N‐myc‐interactor (Nmi), a cytoplasmic protein, enhances STAT1 and STAT5 activation by recruiting them through CBP. The SH2 protein subfamily, which includes the isoforms lymphocyte adapter protein (LnK), SH2‐B, and adapter molecule containing PH and SH2 domains (APS), serves as adapters in the signaling pathway. SH2‐B facilitates glucocorticoid hormone‐induced JAK2 activation, while APS functions as a negative regulator of JAK/STAT signaling. Other negative regulators include the suppressors of cytokine signaling/cytokine‐inducible SH2 protein (CIS) family, protein inhibitor of activated STAT and protein tyrosine phosphatases.^28^

Leptin is a hormone that acts on the hypothalamus to regulate food intake and energy expenditure. The ob/ob mice lacking leptin or the db/db mice lacking the leptin receptor, develop severe obesity and insulin resistance.^29^ When leptin binds to the *β*‐isoform of the leptin receptor, it activates JAK2 through auto‐phosphorylation. This process phosphorylates tyrosine residues on the cytoplasmic tail of the receptors, such as Y985, X1077 and Y1138, enabling the recruitment and phosphorylation of signaling molecules STAT3 and STAT5. The activation of STAT3 increases the expression of genes encoding proopiomelanocortin (POMC) and inhibits the expression of Agouti‐related peptide (AgRP) and neuropeptide Y (NPY) in the neurons of the hypothalamic arcuate nucleus. The POMC and AgRP/NPY peptides have opposite functions and mediate anorectic responses to leptin, promoting changes in satiety.^22^

The targeted knockout of STAT3 in neural tissue, including the hypothalamus, leads to obesity that closely resembles the phenotype of ob/ob or db/db mice.^30^ Deleting STAT5 in the brains of mice also results in obesity, primarily due to increased food intake, indicating that STAT5 activation is crucial for satiety in the central nervous system.^31^ However, the specific STAT5 target genes responsible for this effect have not yet been identified.^31^ Mutations in genes such as those encoding the SH2‐B adapter protein have been linked to aberrant JAK/STAT signaling in the central nervous system, contributing to the development of obesity.^32^, ^33^

In the liver, hepatic steatosis is partially regulated by the JAK/STAT signaling mechanism, which is influenced by cytokines and growth factors. Growth hormone, secreted by somatotropic cells in the anterior pituitary gland, plays a critical role in regulating the production of hepatic insulin‐like growth factor 1 (IGF‐1) through activation of JAK2 and STAT5. Aberrations in the IGF/growth hormone axis have been implicated in obesity development in rodents and humans.^34^ It is hypothesized that low levels of growth hormone cause decreased lipolysis and fatty acid oxidation in adipose tissue, leading to increased hepatic steatosis.^35^ Liver‐specific deletion of STAT5 in mice results in obesity and glucose intolerance. The absence of STAT5 in the liver increases the phosphorylation of STAT1 and expression of its target genes, including CD36, PPAR‐γ, and PGC‐1α/β, respectively, leading to increased lipogenesis, fatty acid uptake, and steatosis. This suggests that loss of STAT5 induces the expression of CD36, PPARγ, and PGC1α/β.^36^ Several studies have emphasized the importance of STAT3 in liver function. Liver‐specific deficiency of STAT3 has been shown to cause insulin resistance and increased expression of gluconeogenic genes. Conversely, activation of STAT3 in liver cells has been found to prevent hepatic lipid accumulation.^37^

### 2.3 Obesity and the PI3K/AKT pathway

The PI3K/AKT pathway is activated by various upstream signals, such as hormones and growth factors, which contribute to obesity. Upon activation, PI3K converts phosphatidylinositol‐4,5‐bisphosphate (PIP2) to phosphatidylinositol‐3,4,5‐triphosphate (PIP3), subsequently activating AKT. AKT then regulates glycogen synthase kinase 3, protein kinase C and the Forkhead‐box (FOX) protein family to control glycogen synthesis, glucose uptake, and adipogenesis. The primary targets of the PI3K/AKT pathway, including the mechanistic target of rapamycin (mTOR) complex 1 (mTORC1) and mTORC2 also play a significant role in the development of obesity.^38^

Research has shown that leptin suppresses food intake partly through the PI3K/AKT/FOXO1 pathway. Inhibition of PI3K specifically reduces the effects of leptin, highlighting the importance of PI3K/AKT in appetite regulation. Additionally, the PI3K/AKT/mTOR signaling pathway has dual effects on thermogenesis and negatively regulates food intake.^39^ One of the most notable impacts of the PI3K/AKT pathway is its role in insulin signaling. Impairment of PI3K/AKT signaling leads to the degradation of sortilin 1 (SORT1), a component of glucose transporter 4 (GLUT4) storage vesicles, and decreases insulin sensitivity.^40^ In the liver, the PI3K/AKT/mTOR and PI3/AKT/FOXO1 pathways contribute to *de novo* lipogenesis and hepatic glucose production.^41^, ^42^

Inflammation of adipose tissue is a key factor in insulin resistance. The infiltration and accumulation of CD4^+^ T cells are primary events that play a crucial role in initiating adipose tissue inflammation. Recently, it was discovered that Kruppel‐like‐zinc finger family 10 (KLF10) in CD4^+^ T cells plays a crucial role in obesity and insulin resistance. These effects are mainly mediated by the PI3K‐AKT‐mTOR signaling pathway.^43^

### 2.4 The role of AMPK pathway in obesity

Recent evidence has illuminated the AMP‐activated protein kinase (AMPK) pathway and its involvement in obesity, insulin resistance, and BAT thermogenesis. In the central nervous system, activation of AMPK contributes to weight gain, and AMPK plays a role in a feedback system that regulates feeding mechanisms. Consequently, fasting boosts AMPK activity in the hypothalamus, while refeeding suppresses its activity.^44^ It is therefore proposed that AMPK levels in adipose tissue are diminished in individuals with insulin resistance, aligning with the finding that mice with an adipocyte‐specific disruption of AMPK are more susceptible to developing insulin resistance and related conditions.^45^ The activation of AMPK in adipocytes inhibits adipogenesis, enhances insulin sensitivity, promotes thermogenesis, and leads to weight loss. However, in the central nervous system, AMPK activation results in insulin resistance, reduced thermogenesis, weight gain, and increased appetite.

### 2.5 Obesity and TGF‐β signaling: An overview

The TGF‐β superfamily, which includes TGF‐β1, TGF‐β2, TGF‐β3, bone morphogenetic proteins and growth/differentiation factors plays a versatile role in the development of obesity.

The pro‐fibrotic, remodeling and pro‐inflammatory functions of TGF‐β mechanistically link to many comorbidities associated with obesity. The TGF‐β family comprises 33 structurally and functionally related growth factors. TGF‐β1 signals downstream through SMAD‐dependent and SMAD‐independent pathways and acts through a heteromeric receptor complex comprising type 1 and type 2 receptors.^46^, ^47^ The cellular effects of TGF‐β are mediated by receptor‐activated SMADs (R‐SMADs). The phosphorylated type 1 receptor phosphorylates the SMAD protein targets with SMAD two‐thirds forming a complex with SMAD4, translocating into the nucleus to regulate gene expression. The SMAD2/3/4 complex is considered a co‐activator, interacting with CBP and p300 to enhance gene expression. In parallel, the SMAD complex interacts with chromatin remodeling complexes in the nucleus to regulate the active chromatin status. SMADs 6 and 7 are the inhibitory SMADs that inhibit SMAD2 and SMAD3 phosphorylation to attenuate complex formation with SMAD4 for transcriptional activity in the nucleus.^48^

TGF‐β1 signaling plays a crucial role in regulating energy homeostasis, insulin resistance and phenotypic switching in BAT and white adipose tissue. It has been demonstrated that obesity increases hepatic TGF‐β1 activity along with markers of inflammation.^49^ In animal models of obesity induced by a high‐fat diet, TGF‐β and plasminogen activator inhibitor‐1 (PAI‐1) mRNA levels were increased in adipose tissue. In murine models of obesity, TGFβ was shown to increase CD206^+^ M2‐like macrophages in white adipose tissue that are linked to decreased white adipose tissue browning in insulin resistance.^50^ In summary, TGF‐β contributes to dysfunctional adipose tissue in obesity with impaired adipogenesis and amplified inflammation and fibrosis. TGF‐β induced adipose tissue progenitor cells to increase the expression of collagen and IL‐6 thereby promoting pro‐fibrotic and inflammatory phenotypes. TGF‐β1 also signals through SMAD‐independent pathways such as MAPKs, PI3K/AKT and Ras‐related C3 botulinium toxin substrate (RAC)/cell division cycle 42 (CDC42).^51^

One important member of the TGF‐β superfamily, GDF‐15, has been identified as a crucial regulator of appetite. It has been reported that mice lacking GDF‐15 exhibit increased adiposity. Another member, BMP‐4, and its antagonist Gremlin, are known to promote white adipose tissue browning, thereby reducing adiposity.^52^, ^53^

TGF‐β signaling is also involved in the regulation of glucose tolerance. Blocking of TGF‐β/SMAD3 signaling has been shown to have protective effects on mice, preventing obesity, insulin resistance, and hepatic steatosis. This protection is achieved through the activation of PPARγ in adipose tissue.^54^

### 2.6 Endoplasmic reticulum signaling pathways and obesity

Recently, there has been an increased focus on obesity‐induced ER stress, where unfolded proteins accumulate in the ER, causing stress and metabolic dysfunction. In conditions of ER stress, a protein called mesencephalic astrocyte‐derived neurotrophic factor (MANF) is released in large amounts and activates the unfolded protein response signaling pathway, while also negatively regulating NF‐κB signaling.^55^ NF‐κB is an inducible factor that has a low constitutive expression but can be strongly induced by a various factors. It acts as a general stress sensor, inducing gene‐regulatory mechanisms, and fine‐tuning mRNA and protein expression of specific sets of genes.^56^ MANF in the hypothalamic regions leads to increased appetite and adiposity by inducing the expression of phosphatidylinositol‐5‐phosphate 4‐kinase type 2 beta (PIP4K2b), which triggers insulin resistance and ultimately results in the accumulation of fat mass.^55^

A few studies have shown that mice with liver‐specific knockout of MANF demonstrate impaired browning of white adipose tissue and exaggerated obesity. However, liver‐specific overexpression of MANF protects these mice against diet‐induced obesity by enhancing WAT browning.^57^ IRE1α, an ER stress sensor activated by nutrients, hormones, and immunological triggers, leads to increased IL‐1β secretion. Other inflammatory triggers such as lipopolysaccharides and IL‐4, activate splicing of IRE1α through interactions with toll‐like receptors.^58^

### 2.7 High blood pressure and obesity

The risk of developing CVDs is significantly higher in hypertensive obese individuals compared to non‐obese individuals. It has been identified that 85% of high blood pressure occurs in patients with a BMI greater than 25 kg/m^2^.^59^ According to the guidelines of the American Heart Association, hypertension is defined as systolic blood pressure ≥130 mmHg and diastolic blood pressure ≥80 mmHg.^60^

The relationship between obesity and hypertension can be explained by several mechanisms, including enhanced renal absorption of sodium, activation of the renin‐angiotensin aldosterone system (RAAS) and sympathetic nervous system (SNS), release of angiotensin from adipose tissue and insulin resistance.^61^ The overactivity of the SNS manifests as an increase in heart rate, elevated cardiac output and renal tubular sodium reabsorption as a direct effect of *α* and *β* adrenergic activation.^62^ It is important to note that this SNS activation is exaggerated even with slight weight gain. The activation of the SNS in obesity is a consequence of abnormal adipokine secretion from adipose tissue, insulin resistance, and stimulation via RAAS.^63^ Moreover, the sympathetic tone due to excess adiposity is influenced by a multitude of factors, such as visceral fat accumulation, ethnicity, and sex differences. The overactivation of the SNS predominantly affects the kidneys and skeletal muscles.^63^

The activation of RAAS leads to higher levels of plasma renin. Angiotensin, produced by the RAAS, induces systemic vasoconstriction and stimulates the production of aldosterone from the adrenal cortex. This results in increased tubular sodium reabsorption and water retention, consequently leading to hypertension.^64^ The activation of RAAS in obesity is attributed to a reciprocal interaction between the sympathetic‐adrenal system and RAAS. Both systems activate each other and drive the upregulation of renin from juxtaglomerular cells. Additionally, excess lipid accumulation causes kidney compression, increasing renin secretion, and excess adipocytes increase angiotensin II production.^65^ One of the notable structural and functional renal changes associated with obesity‐induced hypertension is the accumulation of perirenal fat. This accumulation induces inflammation and expansion of the renal medullary extracellular matrix, compressing the renal medulla.^66^ The resulting decrease in renal tubular blood flow causes a decrease in sodium delivery to the *Macula densa* distally, stimulating a feedback‐mediated reduction in renin secretion. However, the elevated glomerular hydrostatic pressure impairs renal function, exacerbating sodium retention and increasing arterial pressure to maintain sodium delivery to the *macula densa*. Moreover, insulin resistance in obese states contributes to increased blood pressure by directly promoting renal sodium retention in the proximal convoluted tubule through the activation of the sodium‐hydrogen exchanger 3 (NHE3), also known as solute carrier family 9, member 3 (SLC9A3). In obese individuals with chronic hyperinsulinemia, endothelial dysfunction results in vasoconstrictor tone.^59^

It is interesting to note that leptin levels are high in the obese population, indicating a state of leptin resistance. The high levels of leptin contribute to obesity‐induced hypertension through SNS activation.^67^ The treatment of hypertension in obese adults is primarily based on calorie restriction and maintaining a healthy BMI. Although multiple hypertensive medications are available, it is ideal to prefer medications such as angiotensin‐converting enzyme inhibitors, angiotensin II receptor blockers and calcium channel blockers that do not stimulate insulin resistance rather than insulin resistance‐enhancing drugs such as thiazide diuretics and *β*‐blockers.^68^

### 2.8 Obesity and dyslipidemia

The unfavorable shift and changes in the lipid profile of obese individuals include an increase in triglycerides and FFAs, a decrease in HDL, and an increase in LDL. These changes, along with insulin resistance, create a highly atherogenic environment and postprandial hyperlipidemia.^69^ Additionally, there is a shift in lipid metabolism towards the creation of small dense LDL particles.^70^ Obesity, metabolic syndrome, and dyslipidemia are linked by insulin resistance in peripheral tissues, which leads to an increased hepatic flux of fatty acids from lipolysis and from adipose tissue that is resistant to the anti‐lipolytic effects of insulin.^71^, ^72^

The hallmark feature of hypertriglyceridemia, due to increased FFA fluxes leads to hepatic accumulation of triglycerides. This, in turn, increases hepatic synthesis of VLDL hampering the lipolysis of chylomicrons and resulting in increased remnant triglycerides transported to the liver. The reduced activity of lipoprotein lipase in adipose tissue and skeletal muscle further impairs lipolysis.^73^ The increase in chylomicron levels and VLDL levels together with impaired lipolysis affects HDL metabolism.

These dyslipidemic features in obese individuals, along with a pro‐inflammatory gradient, directly affect the endothelium. It is well known that elevated plasma FFA levels are a consequence of FFA release from adipose tissue and impaired clearance of these FFAs. Together with obesity‐induced inflammation, they play a critical role in the development of insulin resistance. Cytotoxic FFAs such as saturated fatty acids, arachidonic acid, and linoleic acids, mediate diet‐induced inflammation, stimulating the synthesis of pro‐inflammatory cytokines such as IL‐1, IL‐6 and TNF‐α.^74^ It is interesting to note that small dense LDL particles are more pro‐atherogenic than large LDL particles due to their decreased affinity to the LDL receptor, causing prolonged circulation time. Strikingly, these particles can enter the arterial wall more easily than large LDL particles and get trapped in the arterial wall by binding to intraarterial proteoglycans. In addition, the small dense LDL particles are more prone to oxidation, resulting in enhanced uptake by macrophages.^75^ Another source of increased fatty acid delivery to the liver is the increase in adipose tissue mass and the increase in fatty acid synthesis by insulin‐stimulated activation of the sterol regulatory element‐binding protein 1c (SREBP1c), a transcription factor that increases the expression of genes involved in fatty acid synthesis.^76^

In cases of insulin resistance in obese individuals, abnormalities in triglyceride lipoprotein metabolism, such as an increase in apolipoprotein C‐III (APOC3) expression, lead to reduced clearance of triglyceride‐rich lipoproteins. APOC3 acts as an inhibitor of lipoprotein lipase (LPL).^77^ Additionally, circulating levels of adiponectin are decreased in obese individuals, resulting in higher serum triglycerides. This is further supported by a study in mice that showed a decrease in triglycerides and an increase in HDL cholesterol in those that overexpressed adiponectin.^78^

The multifactorial pathophysiology of dyslipidemia in obesity includes hepatic overproduction of VLDL, decreased triglyceride lipolysis, impaired peripheral FFA clearance, increased FFA fluxes from adipose tissue to the liver, and the formation of small dense LDL particles. A combination of dietary and lifestyle modifications, such as exercise, has proven beneficial in managing dyslipidemia in obese populations. Weight loss alone has been shown to be effective in decreasing LDL levels and increasing lipoprotein lipase activity. Pharmacological interventions for dyslipidemia primarily involve the use of statins, which are the popular first line of therapy due to their favorable effects on LDL profiles. However, statins have been found to have little effect on triglyceride levels in dyslipidemic obese individuals.^79^ Recently, bariatric surgery‐induced weight loss has gained popularity and has been associated with increased HDL cholesterol, decreased triglyceride levels, and is a therapeutic option if the combination of lifestyle modifications and pharmacotherapy fails.^80^, ^81^

An increase in the release of FFAs from adipose tissue through lipolysis can result in an enhanced delivery of FFAs to the liver. This, in turn, leads to an increased production of triglycerides and VLDL in the liver, as well as the inhibition of lipoprotein lipase in adipose tissue and skeletal muscle. These factors contribute to hypertriglyceridemia. Additionally, the increased VLDL in the liver can hinder the lipolysis of chylomicrons, further exacerbating hypertriglyceridemia. The cholesteryl ester transport protein exchanges triglycerides and VLDL for cholesteryl esters from LDL and HDL, creating triglyceride‐rich LDL and HDL. Hepatic lipase then hydrolyzes the triglycerides in LDL and HDL, resulting in the production of small, dense LDL and HDL.

### 2.9 MASLD and obesity

The accumulation of fat in the liver, known as intrahepatic lipid accumulation, is primarily caused by elevated levels of FFAs, resulting from an increased BMI. In the case of insulin resistance, the rise in FFA production and intrahepatic lipid accumulation occurs because carbohydrate metabolism shifts away from intramuscular glycogen stores and towards the liver.^82^ Together these effects can result in metabolic dysfunction‐associated liver disease (MAFLD) or metabolic dysfunction‐associated steatotic liver disease (MASLD), formerly known as non‐alcoholic fatty liver disease and nonalcoholic steatohepatitis (NASH).^83^ Another connection between MASLD and increased BMI is the heightened expression of hepatic lipase and lipoprotein lipase, which enhances triglyceride metabolism within the liver.^84^ The combination of elevated circulating FFAs and increased intrahepatic triglyceride metabolism contributes to the development and progression of hepatic steatosis. The association between obesity and a constellation of liver abnormalities, characterized by increased hepatic fatty acid uptake and de novo lipogenesis, is mainly characterized by abnormalities in glucose, fatty acids, lipoprotein metabolism and inflammation, leading to increased levels of intrahepatic triglycerides.^85^ In parallel, the alterations in fatty acid transport promote the accumulation of ectopic lipids in the liver of individuals with insulin resistance, leading to an increase in intrahepatic triglyceride content and redirection of plasma FFAs from adipose tissue to other tissues. This is supported by the finding that the expression of CD36, which regulates tissue fatty acid uptake from plasma, is decreased in adipose tissue and increased in the liver and skeletal muscle of obese individuals with insulin resistance.^86^

During *de novo* lipogenesis carbohydrates are converted to fatty acids. In this process several enzymes are important and are primarily regulated at the transcriptional level.^87^ These enzyme accounts for less than 5% of the fatty acids incorporated into secreted very low‐density lipoprotein triglycerides. However, in MASLD, the contribution of de novo lipogenesis to total intrahepatic glucose production is much higher, accounting for 15%–23% of fatty acids secreted in very low‐density lipoprotein triglycerides.^88^ This suggests that in obese individuals with insulin resistance, the rate of de novo lipogenesis may have important regulatory functions. Intrahepatocellular fatty acid oxidation primarily occurs within the mitochondria, and the inhibition or activation of this oxidation can influence lipid accumulation and triglyceride content in the liver. Mitochondrial dysfunction and deficiencies in mitochondrial enzymes lead to hepatic steatosis.^89^

**FIGURE 2:**
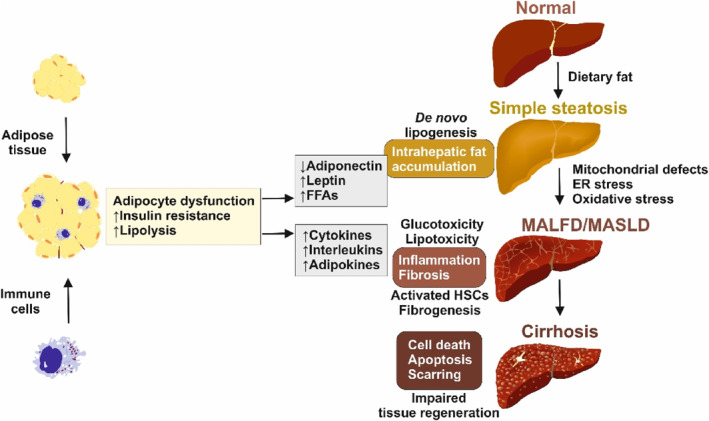
Pathogenetic events leading to metabolic dysfunction‐associated fatty liver disease (MAFLD) and metabolic dysfunction‐associated liver disease (MASLD). Expansion and low‐grade inflammation of adipose tissue are hallmarks of obesity, which result in dysfunction in adipocytes, insulin resistance, and increased rates of lipolysis. Additionally, adipose tissue secretes high levels of cytokines (specifically interleukins), adipokines, leptins, and free fatty acids, while the expression of adiponectin, a factor that regulates glucose levels, decreases. As a result, hepatic *de novo* lipogenesis is stimulated, leading to the accumulation of fat in the liver and lipotoxicity. This, in combination with chronic glucotoxicity, triggers endoplasmic reticulum stress, oxidative stress, mitochondrial defects, cell death, and apoptosis. In liver tissue, quiescent hepatic stellate cells become activated and produce large amounts of extracellular matrix compounds, ultimately resulting in fibrosis and cirrhosis.

Collectively, the interrelationship between steatosis and increased BMI is due to various factors, such as an increased rate of FFA uptake by the liver, increased intrahepatic *de novo* lipogenesis of fatty acids, increased production and secretion of triglycerides in VLDL, and inhibition of fatty acid oxidation by high plasma glucose, which affects SREBP1 and carbohydrate‐responsive element‐binding protein (ChREBP). It is critical to manage MASLD with weight loss and treat contributing conditions, such as DM, before it progresses to cirrhosis, which can cause irreversible damage to the liver. Figure 2 provides a summary of the interactions between increased adiposity and MASLD.

### 2.10 Obesity and gastrointestinal complications

Obesity‐related gastrointestinal complications include gastroesophageal reflux disease (GERD), hepatocellular carcinoma, cholangiocarcinoma, pancreatic cancer, Barrett's esophagus, gallstones, and esophageal cancers.^90^ These gastrointestinal conditions are attributed to insulin resistance, which is caused by increased levels of FFAs, leptin, TNF‐α, resistin, and a decrease in adiponectin. As a result, there is an increase in insulin‐like growth factor‐binding protein 1 and IGFBP2 levels in obese individuals with insulin resistance. This leads to an increase in IGF‐1 bioavailability, which promotes cell proliferation in target cells and inhibits apoptosis. The activation of the IGF receptor signaling pathway caused by increased IGF‐1 further contributes to the development of cancers. Cell proliferation is induced through the activation of PI3K and AKT/PKB, as well as mTOR and RAS/RAF/MAPK pathways.^91^

Adiponectin is an antidiabetic, insulin sensitizer with anti‐inflammatory properties. It inhibits cell proliferation and induces apoptosis through various pathways such as adiponectin receptor (adipoR) 1‐ and adipoR2‐mediated AMPK activation. It also increases expression of AKT and ERK signaling pathways in pancreatic *β*‐cells and lung epithelial cells. Circulating adiponectin has an inverse relationship with BMI,^92^ which leads to an increased risk of gastrointestinal cancers in obese individuals due to decreased adiponectin levels.

Multiple studies have shown that obesity is a significant risk factor for GERD.^93^ Abdominal visceral obesity is closely associated with GERD compared to BMI. This is due to lower esophageal sphincter abnormalities, an increased risk of hiatal hernia, and increased intragastric pressure. The increase in intra‐abdominal pressure causes reflux of gastric contents within the esophageal body, while altered post‐esophageal pressure gradient causes retrograde flow of gastric contents.^94^ In addition, abnormalities in adiponectin and leptin secretion are proposed to link obesity and GERD by modulating gastrointestinal motility both centrally and peripherally.

**FIGURE 3:**
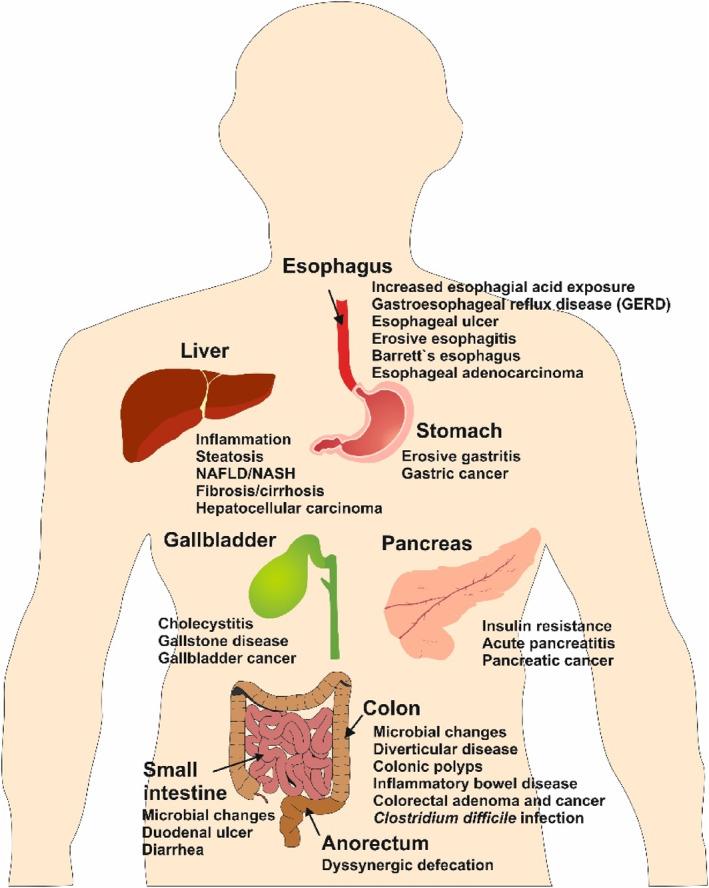
Gastrointestinal and hepatobiliary complications associated with obesity. Excess body weight and obesity are risk factors for various gastrointestinal and hepatobiliary malignancies that can affect the esophagus, stomach, colon, small intestine, anorectum, liver, gallbladder, and pancreas. The symptoms and conditions related to the gastrointestinal system are wide‐ranging. This figure has been modified and expanded from the original work by Camilleri and colleagues.^97^

In the obese population, symptoms of functional dyspepsia, such as bloating, vomiting, nausea and discomfort, are caused by gastric motor dysfunction, impaired gastric emptying, and gastric motility.^95^ The risk of gallbladder disease also increases with obesity. A systematic review of 17 prospective studies involving 1,192,103 participants showed a 2‐fold increase in gallbladder disease in obese individuals.^96^ Gallbladder dysmotility and increased leptin levels due to abdominal obesity and insulin resistance are the suggested mechanisms to explain the relationship between obesity and gallbladder dysfunction.^97^ While weight reduction alone is not a robust treatment for GERD, modest weight loss could help alleviate GERD symptoms. Other management strategies include symptomatic treatment along with weight loss strategies. The gastrointestinal and hepatobiliary complications associated with obesity are depicted in Figure 3.

### 2.11 Obstructive sleep apnea

OSA is a common condition in obese individuals, characterized by excessive daytime sleepiness and an increased risk of CVD. The proposed mechanisms linking obesity and OSA include an increase in upper airway adiposity, elevated levels of adipokines, and an enlarged neck circumference.^98^ It is characterized by the collapse of the upper airways during sleep, resulting in intermittent hypoxia and sleep disruption. This condition is associated with an increased risk of cardiovascular problems. Typically, OSA patients are overweight or obese and often have other conditions such as hypertension, type 2 DM, or dyslipidemia.^99^ The linear correlation between obesity and OSA can be explained by a decrease in muscle activity in the upper respiratory tract due to fat deposits. This leads to a narrowing of the airway, hypoxic episodes, and sleep apnea. These hypoxic episodes result in a decrease in oxygen availability to body tissues and blood vessels contributing to a high risk of CVD.^100^ Obese individuals often experience short sleep duration, which can lead to hormonal imbalances. For instance, a lack of sleep can decrease melatonin levels, disrupting the metabolic circadian rhythm and increasing the risk of metabolic syndrome.^101^ In addition, altered levels of leptin and insulin can increase food craving and contribute to excessive calorie intake, further raising the risk of DM and other complications associated with metabolic syndrome.

**FIGURE 4:**
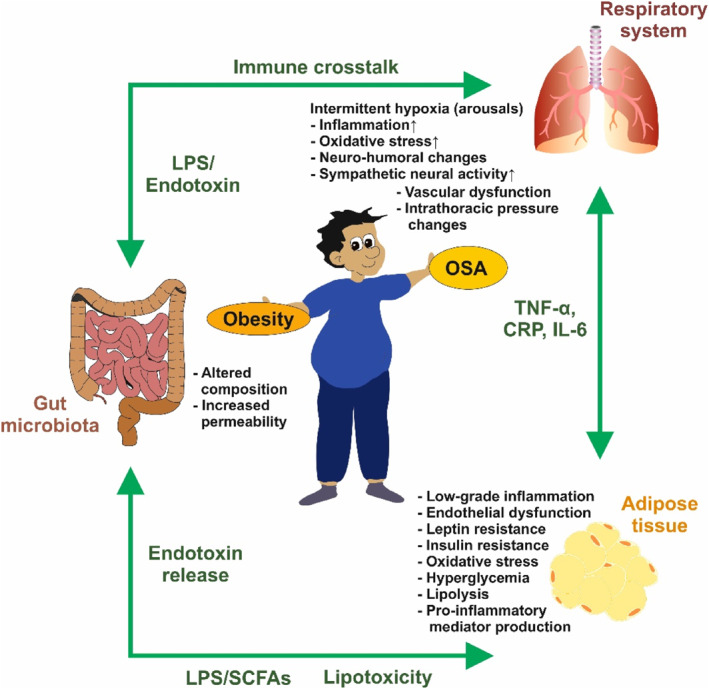
Relationship between obesity and obstructive sleep apnea (OSA) syndrome. Dysfunction of the adipose tissue, gut, and respiratory system are closely related in obesity‐induced OSA. Accumulated fat produces low‐grade inflammation, lipolysis, oxidative stress, hyperglycemia, leptin and insulin resistance, as well as significant pro‐inflammatory mediator production. In parallel, the composition of the gut microbiota is altered, leading to increased gut permeability and release of endotoxins such as lipopolysaccharides (LPS) that affect the adipose tissue and the respiratory system. Moreover, short‐chain fatty acids (SCFAs) generated from the gut microbiota can significantly contribute to the development of metabolic disorders. Overweight further reduces respiratory muscle strength, leading to intermittent hypoxia that further triggers inflammation, oxidative stress, vascular function, and provokes neuro‐humoral changes. Additional information about the relationship between obesity and obstructive sleep apnea and the adverse effects on other tissues are discussed in detail elsewhere.^100^

In the case of OSA, episodes of apnea or hypoxia can trigger increased sympathetic activation and cause a drop in oxyhemoglobin saturation from 95% to 80%. It is important to note that hypoxia can lead to oxidative stress, resulting in the overproduction of reactive oxygen species (ROS). This can lead to endothelial dysfunction and ultimately contribute to the development of atherosclerosis.^102^ Making dietary modifications and increasing physical activity can be effective strategies for weight loss and alleviating symptoms of moderate OSA. Continuous positive airway pressure is the standard treatment for this disease and can improve the functional status of individuals with this condition. It is crucial to take a multi‐targeted approach to address obesity and manage OSA in order to reduce the risk of CVD in this population. Figure 4 provides a visual representation of the relationship between obesity and OSA.

### 2.12 Polycystic ovary syndrome and obesity: A pathophysiological view

Polycystic ovary syndrome (PCOS) is the most common endocrine disorder, affecting around 7% of women in the reproductive age group. It is characterized by increased production of androgens and abnormal levels of gonadotropin hormones. This leads to anovulation, infertility or impaired fertility, menstrual irregularities, acne, and androgenic alopecia. Strikingly, the PCOS population often demonstrates insulin resistance and hirsutism. PCOS is also associated with impaired glucose tolerance, MASLD, dyslipidemia, and OSA. The increased adiposity in PCOS has deleterious effects on hormonal levels, specifically increasing testosterone levels. This leads to central obesity, visceral fat distribution, and the interplay of obesity and hormonal levels negatively impacting fertility.^103^ It is alarming to note that 40%–80% of PCOS patients are obese.^104^

PCOS is closely related to insulin resistance, and it is proposed that testosterone and the CAG repeat number within the androgen receptor contribute to the development of insulin resistance. The post‐receptor defect specific to the PI3K pathways and the stimulating effects on intact MAP kinase pathway by compensatory hyperinsulinemia enhance steroidogenesis.^103^ The adverse effects of hyperinsulinemia on preantral follicular development cause ovulatory dysfunction. Rodent models show the development of hyperandrogenemia with enhancement of luteinizing hormone (LH) pulse amplitude in the pituitary and stimulation of adrenal cytochrome P450C17α activity.^104^ The effects of hyperinsulinemia in insulin resistance drive enhanced steroidogenesis within the ovary and direct effects within the adrenal resulting in hyperandrogenic features.^103^

Adipocytes in women with PCOS exhibit abnormal lipolytic function. Androgens have a stimulatory effect on lipolysis in these adipocytes, leading to impaired adipocyte differentiation, insulin signaling, and adipokines. Therefore, androgen‐mediated enhanced visceral lipolysis is a significant mechanism involved in PCOS.^105^ Increased fat mass amplifies LH stimulation by enhancing the sensitivity of thecal cells, resulting in increased ovarian androgen production. Given the close relationship between PCOS and insulin resistance, it is crucial to understand the underlying mechanism of insulin resistance in PCOS. Visceral fat accumulation plays an essential role in contributing to insulin resistance in PCOS through the effects of adipokines and fatty acid release.^106^ Thus, in addition to its association with the pleotropic steroidogenic effects of excessive insulin through the intact MAPK post‐receptor insulin pathway and impaired PI3K pathway, the core component of PCOS pathogenesis is linked to insulin resistance in obese women. Compensatory hyperinsulinemia also plays an important role in the pathophysiology of the pituitary‐ovarian axis. The management of PCOS revolves around weight loss and the treatment of insulin resistance and anovulation.^105^

Given the complexity of PCOS pathogenesis, it is important to consider the effects of PCOS on further weight gain and hindering weight loss efforts.^107^ As mentioned earlier, PCOS is responsible for abnormalities in the lipolytic functioning of adipocytes. One study demonstrated that catecholamine induced lipolysis was increased twofold within the isolated visceral adipocytes in non‐obese women with PCOS compared with BMI‐matched control women. This increase was possibly mediated by changes in the function of the post‐receptor protein kinase A‐hormone‐sensitive lipase complex.^108^ Although the effects of androgens on adipocytes cause stimulation of lipolysis, impaired adipocyte differentiation, insulin signaling and generation of adipokines, there is tight control of exposure of adipocytes to androgens through key isoenzymes. Thus, the effects of androgens on adipocyte function and lipolysis in PCOS women are incompletely understood and further research is needed.^109^

In addition to features like hirsutism, menstrual irregularities and fertility problems, women with PCOS are also susceptible to mental health problems. This provides a reasonable explanation for the potential hindrance to successful weight loss measures in PCOS women with anxiety and depression. In summary, a vicious cycle may result in worsening PCOS features and weight gain due to hindrances in weight loss measures.^110^, ^111^

As discussed it is generally assumed that obesity is a risk factor for the development of PCOS and the majority of women with PCOS are either overweight or obese. Importantly, insulin resistance and hyperinsulinemia are drivers for enhanced steroidogenesis in women with PCOS, which provokes hyperandrogenaemia and hyperandrogenic features including hirsutism that are regularly seen in women with PCOS. Conversely, PCOS is associated with increased 5‐α reductase activity, which catalyzes the conversion of testosterone into 5‐dihydroxytestosterone acting as a potent androgen and provoking the breakdown of cortisol. This subsequently enhances hypothalamo‐pituitary adrenal axis activity and androgen steroidogenesis.^103^ Therefore, it is reasonable to conclude that obesity and PCOS are, in part, reciprocally linked.

**FIGURE 5:**
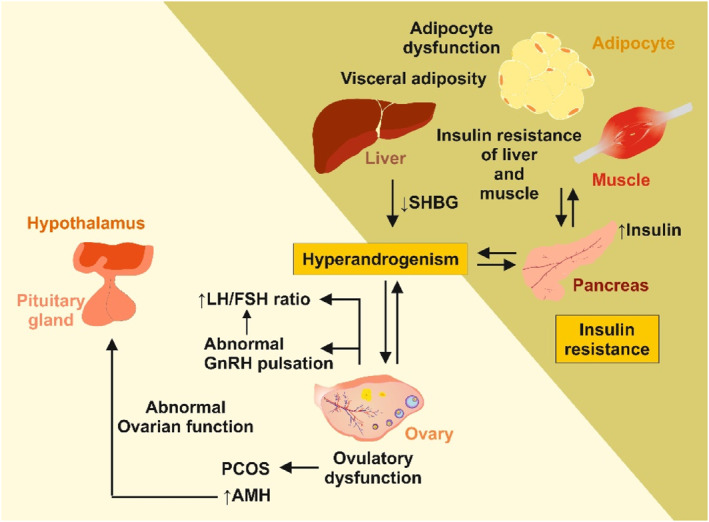
Central events leading to polycystic ovary syndrome (PCOS). Insulin resistance and hyperandrogenism are two synergistic factors that trigger PCOS. The elevated secretion of androgens is caused by intrinsic dysfunction of the ovarian theca cells and dysfunction of the hypothalamus‐pituitary gland‐ovarian axis. In obese individuals, insulin resistance and hyperinsulinemia alters the pulsation of the pituitary gland, leading to disorderly release of gonadotropin‐releasing hormone (GnRH), follicle‐stimulating hormone (FSH), and luteinizing hormone (LH). The elevated ratio of LH to FSH in females results in a dysfunctional menstrual cycle, which is a hallmark of PCOS. Additionally, in the early stage of development, there is an increased follicular mass or hypersecretion by follicles, leading to elevated levels of Anti‐Müllerian hormone (AMH). AMH can be used as a strong diagnostic predictor for PCOS. Interestingly, obese individuals typically have decreased circulating levels of sex hormone‐binding globulin (SHBG) produced by the liver, which is also observed in patients with PCOS. For more details about the pathophysiology of PCOS and factors influencing this endocrine disorder, recent publications provide further information.^149^, ^150^

The complex interaction between increased adiposity and PCOS is depicted in Figure 5.

### 2.13 The intertwined elements of obesity and type 2 diabetes

The term ‘diabesity‘ describes the simultaneous presence of obesity and type 2 DM and their interconnected relationship, as the risk of type 2 DM increases with higher BMI. The relationship between DM and obesity has been the focus of numerous studies due to the concurrent rise in the prevalence of obesity and type 2 DM worldwide. The pathophysiological mechanisms include adiposity‐induced alterations in β‐cell and insulin resistance in multiple organs. A significant mechanism linking obesity and DM is the increase in visceral fat caused by the transportation of excess hepatic triglycerides in VLDL to various tissues, including the β‐cells of the pancreas, ultimately resulting in β‐cell dysfunction and type 2 DM.^112^

Excess body fat, especially intraabdominal fat, is responsible for a multitude of metabolic abnormalities such as increased plasma triglycerides, low HDL cholesterol and beta cell dysfunction. The increase in basal and postprandial plasma insulin levels in obese individuals is caused by increased pancreatic insulin secretion and decreased clearance of portal and peripheral plasma insulin.^85^ β‐cell dysfunction is primarily caused by elevated plasma FFAs and other lipid mediators, such as ceramides and diacylglycerols.

Due to nutrition overload and adiposity, adipose tissue, whose function is to maintain energy balance during fasting and feeding, expands and its mass increases due to the accumulation of triglycerides in adipocytes.^114^ Adipocyte hypoxia due to oxygen demand and inadequate oxygen delivery stimulates adipose tissue fibrogenesis and macrophage chemotaxis, Adipose tissue pro‐inflammatory cytokines, decreased adiponectin, increased lipolysis and the release of FFAs are factors contributing to whole‐body insulin resistance.^115^, ^116^, ^117^, ^118^

Interestingly, individuals with obesity have an impaired ability of insulin to suppress hepatic glucose production.^119^ Over the years, the activation of inflammatory pathways has been implicated in the pathogenesis of insulin resistance in the obese population. Inflammatory pathways such as IκB kinase (IKKB) and JNK1 are activated by TNF‐α, FFAs, diacylglycerols, ceramides, and ROS.^120^ In obesity, contributors to chronic inflammation include ER stress, decreased adiponectin, increased leptin, macrophage infiltration and lipolysis.^120^ Furthermore, the ectopic accumulation of fat in muscles and adipose tissue in obese individuals causes mitochondrial dysfunction and impaired mitochondrial oxidative activity and ATP synthesis. Obesity also accelerates aging of adipose tissue, increases ROS formation in adipocytes resulting in impaired glucose tolerance and IR. Lastly, impaired translocation of GLUT4 has been implicated in the development of IR in the obese population.^121^

In summary, a combination of endocrine, neural, and inflammatory factors link obesity to type 2 DM. A holistic approach is necessary to target these defective signaling pathways to effectively manage type 2 DM.

### 2.14 Obesity – A hallmark feature of cardiovascular disease

Multiple mechanisms have been proposed on how obesity drives structural, functional, and hemodynamic alterations in the development of coronary artery disease, heart failure and arrhythmias. Obesity has a stronger relationship with ischemic stroke and coronary heart disease. Unhealthy dietary habits and lack of physical activity result in ectopic lipid accumulation and metabolic abnormalities such as dyslipidemia and insulin resistance. These, in turn, increase the risk factors for CVD such as type 2 DM, hypertension, and atherogenesis. In the case of PCOS, the threat of CVD increases due to various triggering factors such as OSA, hypertension, metabolic dysfunction‐associated fatty liver disease (MAFLD), and dyslipidemia.^122^ Visceral fat accumulation promotes adipokine expansion, which, in turn, enhances the recruitment and proliferation of pro‐inflammatory macrophages leading to oxidative stress.^123^ Collectively, the aforementioned risk factors are direct risk factors for CVD in obesity.

Obesity or being overweight causes an increase in cardiac output and total blood volume, which contributes to both structural and functional changes in the heart. This increase in intravascular volume leads to left ventricular hypertrophy and left ventricular diastolic dysfunction, making individuals more susceptible to heart failure.^124^ Additionally, the accumulation of fat around the kidneys can compress them, resulting in the upregulation of the RAAS and SNS. This can lead to hypertension and stroke, further worsening heart failure and reducing ejection fraction.^125^ Another indirect association between obesity and CVD is the impairment of physical activity and the presence of musculoskeletal comorbidities such as osteoarthritis. These factors can contribute to further weight gain and escalate the risk of developing CVD.^126^ Various cardiovascular abnormalities are associated with fat mass disorders. These include increased heart rate, left atrial abnormalities, and hemodynamic changes such as increased stroke volume, cardiac output, arterial pressure, and left ventricular stiffness. Structural changes including myocardial fibrosis, left atrial enlargement, left ventricular hypertrophy, right ventricular hypertrophy, and increased pericardial adiposity, are also observed.^124^, ^127^ Additionally, multiple functional changes have been implicated, such as atherosclerosis, thrombosis, myocardial ischemia, deep vein thrombosis, and pulmonary embolism.

Yet another indirect factor is the increased risk of metabolic syndrome, coronary artery disease, and heart failure in individuals with OSA.^128^ Furthermore, the RAAS and SNS in individuals with chronic kidney disease can both elevate the risk of CVD in obese individuals. This risk is influenced by inflammation, dyslipidemia, and hypertension, as well as other factors like coronary calcification, coagulation activation, and endothelial dysfunction.^129^ The primary cause of this strong association is the accumulation of ectopic visceral fat, which promotes chronic inflammation and affects various stages of CVD, including atherosclerosis and thrombosis.^130^

Endothelial dysfunction, linked to CVD, is induced by perivascular adiposity in obese individuals. This adiposity fosters local inflammation and hampers endothelial function.^131^ The malfunction of adipose tissue results in low‐grade inflammation, facilitating immune cell infiltration and leading to insulin resistance, ER stress, and increased ROS production.^132^ The inflammatory progression of atherosclerosis causes coronary calcification, which is notably more prevalent in individuals with abdominal obesity.^133^ Pro‐inflammatory cytokines boost ROS production and activate redox‐sensitive intracellular pathways, increasing the expression of pro‐atherogenic genes, such as heightened expression of adhesion molecules like vascular cell adhesion molecule 1 on endothelial cell surfaces, promoting monocyte infiltration into the subendothelial space.^134^ In summary, the expansion of visceral adipose tissue causes dysregulation of adipokine secretion and increased production of inflammatory cytokines that contribute to insulin resistance, endothelial dysfunction, and a pro‐thrombotic state, ultimately increasing the risk of CVD in obese individuals.

### 2.15 Obesity and cancer

The obesogenic environment is highly complex consisting of alterations in glucose, leptin, adiponectin, glucagon, insulin, cholesterol, and FFAs. Recently, the molecular and cellular processes by which the obese environment affects tumor initiation and progression have been gaining attention. Genetic and non‐genetic alterations govern the interactions between obesity and cancer. In obese states, increased triglycerides cause adipose tissue hypertrophy and hyperplasia, contributing to changes in micro and macro environments.^135^

The satiety‐promoting effects of leptin are impaired by cellular leptin resistance in obesity. In addition to decreased energy expenditure, hyperleptinemia has peripheral effects on cancer cells and the tumor microenvironment, particularly helper T cells. For example, leptin acts as a growth‐stimulating agent in breast cancer, repressing apoptotic pathways, promoting proliferation, modulating metabolic reprogramming and ROS production. Additionally, leptin is associated with cancer stem cell enrichment and epithelial to mesenchymal transition. Interestingly, leptin controls stem cell phenotype through epigenetic mechanisms controlled by the leptin‐STAT3‐G9a histone methyltransferase signaling axis.^136^

Dysregulation of adiponectin has been implicated in colon, liver, renal and pancreatic cancers. This hormone is an insulin sensitizer in the liver and muscle, balancing glucose and lipid metabolism. Adiponectin stimulates ceramidase activity through AdipoR1 and R2 enhancing pro‐apoptotic ceramide catabolism leading to the formation of its downstream anti‐apoptotic metabolite sphingosine‐1‐phosphate S1P.^137^ Strikingly, increased levels of obesity related adipokines such as IL‐6, IL‐8, and TNF‐α are associated with an increased cancer risk. In mouse models, it was reported that a high‐fat diet induced increased FFAs promote PPARγ signaling which is an upstream regulator of WW domain‐containing transcription regulator expression.^138^

Insulin and IGF‐1 activate the INSR and the Insulin growth factor–1 receptor (IGF‐1R), leading to the stimulation of cell proliferation and protein synthesis pathways for tumorigenesis PI3K/AKT/mTOR signaling pathway and RAS‐MAPK pathways.^139^ In obese states, adipocytes in expanding adipose tissue, deposit altered amounts of extracellular matrix (ECM) components, causing ECM remodeling and changes in tissue stiffness. These alterations promote the tumorigenic potential of premalignant breast epithelial cells. Another interesting finding in obese states is the abundant production of collagen VI by the adipocytes.^140^ This proves that dysregulated single extracellular matrix component can promote tumorigenesis.^141^ In intestinal cancers, increased FFAs activate PPARγ‐dependent signaling to promote tumor initiating capacity. In addition to tumor initiation, increased FFAs and fatty acid binding proteins are associated with increased cancer progression. For example, in ovarian cancer, metastasis of cancer cells is fueled by fatty acids delivered by FABP4 from adipocytes.^142^ Furthermore, this metastasis is also associated with STAT3/ALDH1 signaling in these cancers. In breast cancer cells, fatty acids activate mTOR and MAPK signaling to facilitate increased glycolytic and aerobic respiration.^143^ Increased FFAs drive the polarization of adipose tissue macrophages towards a metabolically activated phenotype. This, in turn, alters the niche to support breast cancer stemness and tumorigenesis through the IL‐6/gp130 signaling axis.^144^ In summary, the complexity of the obese environment translates into complex molecular drivers of tumors. Further research might unravel potential therapeutic approaches.

### 2.16 Emerging advances in GLP‐1 targeting obesity

GLP‐1 agonists have recently gained attention for treating obesity and type 2 diabetes. These drugs are designed to mimic endogenous GLP‐1, an incretin hormone produced by L cells in the intestine. GLP‐1 levels rise after meals, promoting insulin production and release, reducing glucagon release, slowing gastric emptying, and increasing satiety. Between 2012 and 2014, three extended‐release GLP‐1 agonists that act centrally were approved for long‐term weight management. However, they were quickly cleared by the kidneys due to their short half‐life. To improve the pharmacokinetic profile of GLP‐1, an approach was developed to create a GLP‐1 analogue resistant to degradation by DPP4, an enzyme that breaks down GLP‐1 quickly.^145^

In Liraglutide, structural modifications were made to GLP‐1 to extend its half‐life to 13 h.^146^ Similarly, in Semaglutide, Lys26 was attached to a hydrophobic C‐18 fatty di‐acid moiety to inhibit glomerular filtration. Tirzepatide, developed in 2022 is a dual‐acting GLP‐RA and GIP‐RA, combining the structural features of GLP‐1 and exenatide.^147^

Liraglutide was the first daily injectable GLP1‐RA approved for type 2 diabetes. The next step was the extensive study of Semaglutide in STEP clinical trials. Semaglutide 2.4 mg subcutaneous injections once a week was approved by the FDA for chronic weight management in June 2021.^148^ Table 1 summarizes the most recent clinical trials on Liraglutide, Semaglutide, and Tirzepatide, and their weight loss outcomes.

**TABLE 1:** Representative clinical studies using GLP‐1 agonist to target overweight.

Study name	Study details	Outcomes	Reference
LEAD 5	Liraglutide 1.8 mg/day (*n* = 232), placebo (*n* = 115) and open label insulin glargine (*n* = 234) were used in combination with metformin 1 g twice per day and glimepiride 4 mg/day for a period of 26 weeks.	The average weight reduction was 1.8 kg in the liraglutide group compared to 0.42 kg in the placebo group.	^151^
LEAD 6	Liraglutide 1.8 mg per day (*n* = 233) or exenatide 10 μg twice per day (*n* = 231) for 26 weeks.	The average weight loss was 3.24 kg in the liraglutide group and 2.78 kg in the exenatide group.	^152^
STEP 6	Semaglutide 2.4 mg or 1.7 mg per week versus placebo in Asian individuals (*n* = 401) for a duration of 20 weeks.	Weight reduction was observed at 13% for 2.4 mg of Semaglutide, 9.6% for 1.7 mg of Semaglutide, and 2.1% for the placebo.	^153^
STEP 8	Semaglutide 2.4 mg versus liraglutide 1.8 mg per day. The study included 331 participants and lasted for 68 weeks	Weight reduction was achieved by 15.8% with Semaglutide and 6.4% with liraglutide	^154^
Surpass −5	Tirzepatide was administered at doses of 5, 10 or 15 mg per week to individuals with type 2 diabetes mellitus who were taking insulin glargine, with or without metformin. The study involved 475 participants and lasted for 40 weeks.	Body weight reduction by 6.2, 8.2, and 10.0 kg respectively	^155^
Surmount 1	Tirzepatide was administered at doses of 5, 10 or 15 mg per week in obese individuals without diabetes. The study included a total of 2539 participants over a period of 72 weeks.	Weight reduction was observed at 15%, 19.5%, and 20.9% in dosages of 5 mg, 10 mg, and 15 mg, respectively, compared to a 3.1% reduction in the placebo group.	^156^

## 3 CONCLUSION

Obesity increases the risk of several debilitating diseases, such as hypertension, CVDs, MAFLD, type 2 DM, and certain cancers. The population that is overweight or obese is at the highest risk of developing CVD and its associated complications. Additionally, obesity itself is an independent risk factor for CVDs. Obesity can cause complications in the upper and lower gastrointestinal tract due to increased intra‐abdominal pressure, including an elevated risk of esophageal cancers. Obesity is the most common risk factor for type 2 DM, which triggers inflammatory pathways, elevated levels of FFAs, adipose tissue hypoxia, and fat accumulation in ectopic locations. Treating obesity is crucial in managing and addressing type 2 DM. The main mechanisms that explain the connection between obesity and high blood pressure involve the activation of the SNS and the RAAS in obese individuals. Excessive fat deposits in the upper respiratory tract of obese individuals can narrow the airway and lead to episodes of hypoxia, resulting in sleep apnea. The majority of women with PCOS are obese, and this condition presents as ovulatory dysfunction, insulin resistance, impaired fertility, hormonal imbalances, and menstrual irregularities. Currently it is discussed, if PCOS and obesity are reciprocally linked. Immediate attention is needed to understand the signaling mechanisms and interactions between obesity and its complications in order to address this complex disease and reduce the global healthcare burden. By unraveling the cellular and molecular signaling network, and gaining a deeper understanding of the cross talk between obesity and the signaling pathways involved in its complications, we can achieve precision medicine in the fight against obesity and its associated co‐morbidities.

## AUTHOR CONTRIBUTIONS

Preethi Chandrasekaran: Conceptualization; data curation; writing – original draft preparation; writing – review & editing. Ralf Weiskirchen: Resources; data curation; writing – original draft preparation; writing – review & editing; visualization; supervision; funding acquisition. All authors have read and agreed to the published version of the manuscript.

## CONFLICT OF INTEREST STATEMENT

The authors declare that they have no competing interests.

## ETHICS STATEMENT

Not applicable.

## Data Availability

In this review no new experimental data is presented.

## References

[1] Cecchini, M. 2018. “Use of Healthcare Services and Expenditure in the US in 2025: The Effect of Obesity and Morbid Obesity.” PLoS One 13(11): e0206703. 10.1371/journal.pone.0206703. 30403716 PMC6221341

[2] Abdelaal, M. , C. W. le Roux , and N. G. Docherty . 2017. “Morbidity and Mortality Associated with Obesity.” Annals of Translational Medicine 5(7): 161. 10.21037/atm.2017.03.107. 28480197 PMC5401682

[3] Arif, M. , D. K. Gaur , N. Gemini , Z. A. Iqbal , and A. H. Alghadir . 2022. “Correlation of Percentage Body Fat, Waist Circumference and Waist‐To‐Hip Ratio with Abdominal Muscle Strength.” Healthcare 10(12): 2467. 10.3390/healthcare10122467. 36553991 PMC9778235

[4] Hassan, M. , N. Ahmad , S. M. Adam , A. Nawi , and H.F. Ghazi . 2016. “Abdominal Obesity Indicators: Waist Circumference or Waist‐To‐Hip Ratio in Malaysian Adults Population.” International Journal of Preventive Medicine 7(1): 82. 10.4103/2008-7802.183654. 27330688 PMC4910307

[5] NCD Risk Factor Collaboration (NCD‐RisC) . 2016. “Trends in Adult Body‐Mass Index in 200 Countries from 1975 to 2014: a Pooled Analysis of 1698 Population‐Based Measurement Studies with 19·2 Million Participants.” Lancet 387: 1377–1396. 10.1016/S0140-6736(16)30054-X. 27115820 PMC7615134

[6] Aune, D. , A. Sen , M. Prasad , T. Norat , I. Janszky , S. Tonstad , P. Romundstad , and L. J. Vatten . 2016. “BMI and All Cause Mortality: Systematic Review and Non‐linear Dose‐Response Meta‐Analysis of 230 Cohort Studies with 3.74 Million Deaths Among 30.3 Million Participants.” BMJ 353: i2156. 10.1136/bmj.i2156. 27146380 PMC4856854

[7] Spalding, K. L. , S. Bernard , E. Näslund , M. Salehpour , G. Possnert , L. Appelsved , K.‐Y. Fu , et al. 2017. “Impact of Fat Mass and Distribution on Lipid Turnover in Human Adipose Tissue.” Nature Communications 8(1): 15253. 10.1038/ncomms15253. PMC5457499 28534500

[8] Kinlen, D. , D. Cody , and D. O'Shea . 2017. “Complications of Obesity.” QJM 111(7): 437–443. 10.1093/qjmed/hcx152. 29025162

[9] Chandrasekaran, P. , and R. Weiskirchen . 2024. “Diabetes Mellitus and Heart Disease.” Metabolism and Target Organ Damage 4(2): 18. 10.20517/mtod.2024.15.

[10] Wen, X. , B. Zhang , B. Wu , H. Xiao , Z. Li , R. Li , X. Xu , and T. Li . 2022. “Signaling Pathways in Obesity: Mechanisms and Therapeutic Interventions.” Signal Transduction and Targeted Therapy 7(1): 298. 10.1038/s41392-022-01149-x. 36031641 PMC9420733

[11] Kassouf, T. , and G. Sumara . 2020. “Impact of Conventional and Atypical MAPKs on the Development of Metabolic Diseases.” Biomolecules 10(9): 1256. 10.3390/biom10091256. 32872540 PMC7563211

[12] Zeng, X. , X. Du , J. Zhang , S. Jiang , J. Liu , Y. Xie , W. Shan , G. He , Q. Sun , and J. Zhao . 2018. “The Essential Function of CARD9 in Diet‐Induced Inflammation and Metabolic Disorders in Mice.” Journal of Cellular and Molecular Medicine 22(6): 2993–3004. 10.1111/jcmm.13494. 29575791 PMC5980191

[13] Hsu, Y.‐M. S. , Y. Zhang , Y. You , D. Wang , H. Li , O. Duramad , X.‐F. Qin , C. Dong , and X. Lin . 2007. “The Adaptor Protein CARD9 Is Required for Innate Immune Responses to Intracellular Pathogens.” Nature Immunology 8(2): 198–205. 10.1038/ni1426. 17187069

[14] Wang, Q. , D. Li , G. Cao , Q. Shi , J. Zhu , M. Zhang , H. Cheng , et al. 2021. “IL‐27 Signalling Promotes Adipocyte Thermogenesis and Energy Expenditure.” Nature 600(7888): 314–318. 10.1038/s41586-021-04127-5. 34819664

[15] Fujishiro, M. , Y. Gotoh , H. Katagiri , H. Sakoda , T. Ogihara , M. Anai , Y. Onishi , et al. 2003. “Three Mitogen‐Activated Protein Kinases Inhibit Insulin Signaling by Different Mechanisms in 3T3‐L1 Adipocytes.” Molecular Endocrinology 17(3): 487–497. 10.1210/me.2002-0131. 12554784

[16] Leonardini, A. , L. Laviola , S. Perrini , A. Natalicchio , and F. Giorgino . 2009. “Cross‐talk between PPARgamma and Insulin Signaling and Modulation of Insulin Sensitivity.” PPAR Research 2009: 818945. 10.1155/2009/818945. 20182551 PMC2826877

[17] Han, J. , J. Wu , and J. Silke . 2020. “An Overview of Mammalian P38 Mitogen‐Activated Protein Kinases, Central Regulators of Cell Stress and Receptor Signaling.” F1000Research 9: 653: F1000 Faculty Rev‐653. 10.12688/f1000research.22092.1. PMC7324945 32612808

[18] Matesanz, N. , I. Nikolic , M. Leiva , M. Pulgarín‐Alfaro , A. M. Santamans , E. Bernardo , A. Mora , et al. 2018. “p38α Blocks Brown Adipose Tissue Thermogenesis through P38δ Inhibition.” PLoS Biology 16(7): e2004455. 10.1371/journal.pbio.2004455. 29979672 PMC6051667

[19] Zhang, S. , H. Cao , Y. Li , Y. Jing , S. Liu , C. Ye , H. Wang , et al. 2018. “Metabolic Benefits of Inhibition of P38α in White Adipose Tissue in Obesity.” PLoS Biology 16(5): e2004225. 10.1371/journal.pbio.2004225. 29750781 PMC5965899

[20] Zhang, X. , L. Fan , J. Wu , H. Xu , J. Wu , K. Fu , J. Wu , et al. 2019. “Macrophage P38α Promotes Nutritional Steatohepatitis through M1 Polarization.” Journal of Hepatology 71(1): 163–174: Erratum in: J Hepatol. 2020;73(3):742‐743. 10.1016/j.jhep.2019.03.014. 32690378

[21] Bak, E.‐J. , K.‐C. Choi , S. Jang , G.‐H. Woo , H. Yoon , Y. Na , Y.‐J. Yoo , Y. Lee , Y. Jeong , and J.‐H. Cha . 2016. “Licochalcone F Alleviates Glucose Tolerance and Chronic Inflammation in Diet‐Induced Obese Mice through Akt and P38 MAPK.” Clinical Nutrition 35(2): 414–421. 10.1016/j.clnu.2015.03.005. 25823386

[22] Zhang, J. , Y. Zhou , C. Chen , F. Yu , Y. Wang , J. Gu , L. Ma , and G. Ho . 2015. “ERK1/2 Mediates Glucose‐Regulated POMC Gene Expression in Hypothalamic Neurons.” Journal of Molecular Endocrinology 54(2): 125–135. 10.1530/JME-14-0330. 25624461

[23] Khoubai, Fatma Zohra , and Christophe F. Grosset . 2021. “DUSP9, a Dual‐Specificity Phosphatase with a Key Role in Cell Biology and Human Diseases.” International Journal of Molecular Sciences 22(21): 11538. 10.3390/ijms222111538. 34768967 PMC8583968

[24] Solinas, G. , and B. Becattini . 2017. “JNK at the Crossroad of Obesity, Insulin Resistance, and Cell Stress Response.” Molecular Metabolism 6(2): 174–184. 10.1016/j.molmet.2016.12.001. 28180059 PMC5279903

[25] Wang, Z. , M. Zhu , M. Wang , Y. Gao , C. Zhang , S. Liu , S. Qu , Z. Liu , and C. Zhang . 2021. “Integrated Multiomic Analysis Reveals the High‐Fat Diet Induced Activation of the MAPK Signaling and Inflammation Associated Metabolic Cascades via Histone Modification in Adipose Tissues.” Frontiers in Genetics 12: 650863. 10.3389/fgene.2021.650863. 34262592 PMC8273343

[26] Corry, J. , H. R. Mott , and D. Owen . 2020. “Activation of STAT Transcription Factors by the Rho‐Family GTPases.” Biochemical Society Transactions 48(5): 2213–2227. 10.1042/BST20200468. 32915198 PMC7609038

[27] Gurzov, E. N. , W. J. Stanley , E. G. Pappas , H. E. Thomas , and D. J. Gough . 2016. “The JAK/STAT Pathway in Obesity and Diabetes.” FEBS Journal 283(16): 3002–3015. 10.1111/febs.13709. 26972840

[28] Hu, X. , J. li , M. Fu , X. Zhao , and W. Wang . 2021. “The JAK/STAT Signaling Pathway: from Bench to Clinic.” Signal Transduction and Targeted Therapy 6(1): 402. 10.1038/s41392-021-00791-1. 34824210 PMC8617206

[29] Wang, B. , P. Chandrasekera , and J. Pippin . 2014. “Leptin‐ and Leptin Receptor‐Deficient Rodent Models: Relevance for Human Type 2 Diabetes.” Current Diabetes Reviews 10(2): 131–145. 10.2174/1573399810666140508121012. 24809394 PMC4082168

[30] Gao, Q. , M. J. Wolfgang , S. Neschen , K. Morino , T. L. Horvath , G. I. Shulman , and X.‐Y. Fu . 2004. “Disruption of Neural Signal Transducer and Activator of Transcription 3 Causes Obesity, Diabetes, Infertility, and Thermal Dysregulation.” Proceedings of the National Academy of Sciences of the U S A 101(13): 4661–4666. 10.1073/pnas.0303992101. PMC384803 15070774

[31] Lee, Ji‐Y. , H. Muenzberg , O. Gavrilova , J. A. Reed , D. Berryman , E. C. Villanueva , G. W. Louis , et al. 2008. “Loss of Cytokine‐STAT5 Signaling in the CNS and Pituitary Gland Alters Energy Balance and Leads to Obesity.” PLoS One 3(2): e1639. 10.1371/journal.pone.0001639. 18286195 PMC2237899

[32] Thorleifsson, G. , G. B. Walters , D. F. Gudbjartsson , V. Steinthorsdottir , P. Sulem , A. Helgadottir , U. Styrkarsdottir , et al. 2009. “Genome‐wide Association Yields New Sequence Variants at Seven Loci that Associate with Measures of Obesity.” Nature Genetics 41(1): 18–24. 10.1038/ng.274. 19079260

[33] Ren, D. , M. Li , C. Duan , and L. Rui . 2005. “Identification of SH2‐B as a Key Regulator of Leptin Sensitivity, Energy Balance, and Body Weight in Mice.” Cell Metabolism 2(2): 95–104. 10.1016/j.cmet.2005.07.004. 16098827

[34] Romero, C. J. , Y. Ng , R. M. Luque , R. D. Kineman , L. Koch , J. C. Bruning , and S. Radovick . 2010. “Targeted Deletion of Somatotroph Insulin‐like Growth Factor‐I Signaling in a Cell‐specific Knockout Mouse Model.” Molecular Endocrinology 24(5): 1077–1089. 10.1210/me.2009-0393. 20211984 PMC2870932

[35] Lichanska, A. M. , and M. J. Waters . 2008. “How Growth Hormone Controls Growth, Obesity and Sexual Dimorphism.” Trends in Genetics 24(1): 41–47. 10.1016/j.tig.2007.10.006. 18063438

[36] Barclay, J. L. , C. N. Nelson , M. Ishikawa , L. A. Murray , L. M. Kerr , T. R. McPhee , E. E. Powell , and M. J. Waters . 2011. “GH‐Dependent STAT5 Signaling Plays an Important Role in Hepatic Lipid Metabolism.” Endocrinology 152(1): 181–192. 10.1210/en.2010-0537. 21084450

[37] Wei, C.‐C. , K. Wu , Y. Gao , L.‐H. Zhang , D.‐D. Li , and Z. Luo . 2017. “Magnesium Reduces Hepatic Lipid Accumulation in Yellow Catfish (*Pelteobagrus fulvidraco*) and Modulates Lipogenesis and Lipolysis via PPARA, JAK‐STAT, and AMPK Pathways in Hepatocytes1.” Journal of Nutrition 147(6): 1070–1078. 10.3945/jn.116.245852. 28424262

[38] Deleyto‐Seldas, N. , and A. Efeyan . 2021. “The mTOR–Autophagy axis and the Control of Metabolism.” Frontiers in Cell and Developmental Biology 9. 10.3389/fcell.2021.655731. PMC8281972 34277603

[39] Kwon, O. , K. W. Kim , and M.‐S. Kim . 2016. “Leptin Signalling Pathways in Hypothalamic Neurons.” Cellular and Molecular Life Sciences 73(7): 1457–1477. 10.1007/s00018-016-2133-1. 26786898 PMC11108307

[40] Zhong, X. , C. Ke , Z. Cai , H. Wu , Y. Ye , X. Liang , L. Yu , et al. 2020. “LNK Deficiency Decreases Obesity‐Induced Insulin Resistance by Regulating GLUT4 through the PI3K‐Akt‐AS160 Pathway in Adipose Tissue.” Aging (Albany NY) 12(17): 17150–17166. 10.18632/aging.103658. 32911464 PMC7521507

[41] Titchenell, P. M. , W. J. Quinn , M. Lu , Q. Chu , W. Lu , C. Li , H. Chen , et al. 2016. “Direct Hepatocyte Insulin Signaling Is Required for Lipogenesis but Is Dispensable for the Suppression of Glucose Production.” Cell Metabolism 23(6): 1154–1166. 10.1016/j.cmet.2016.04.022. 27238637 PMC4909537

[42] Chandrasekaran, P. , and R. Weiskirchen . 2024. “Cellular and Molecular Mechanisms of Insulin Resistance.” Curr Tissue Microenvironment Rep. 10.1007/s43152-024-00056-3.

[43] Wara, A. K. , S. Wang , C. Wu , F. Fang , S. Haemmig , B. N. Weber , C. O. Aydogan , et al. 2020. “KLF10 Deficiency in CD4(+) T Cells Triggers Obesity, Insulin Resistance, and Fatty Liver.” Cell Reports 33(13): 108550. 10.1016/j.celrep.2020.108550. 33378664 PMC7816773

[44] López, M. , R. Nogueiras , M. Tena‐Sempere , and C. Diéguez . 2016. “Hypothalamic AMPK: a Canonical Regulator of Whole‐Body Energy Balance.” Nature Reviews Endocrinology 12(7): 421–432. 10.1038/nrendo.2016.67. 27199291

[45] Desjardins, E. M. , and G. R. Steinberg . 2018. “Emerging Role of AMPK in Brown and Beige Adipose Tissue (BAT): Implications for Obesity, Insulin Resistance, and Type 2 Diabetes.” Current Diabetes Reports 18(10): 80. 10.1007/s11892-018-1049-6. 30120579

[46] Weiskirchen, R. , and S. K. Meurer . 2007. “Bone Morphogenetic Protein‐7 in Focus: a Member of the Transforming Growth Factor‐Beta Superfamily Is Implicated in the Maintenance of Liver Health.” Hepatology 45(5): 1324–1325. 10.1002/hep.21694. 17464975

[47] Miyazawa, K. , Y. Itoh , H. Fu , and K. Miyazono . 2024. “Receptor‐activated Transcription Factors and beyond: Multiple Modes of Smad2/3‐dependent Transmission of TGF‐β Signaling.” Journal of Biological Chemistry 300(5): 107256. 10.1016/j.jbc.2024.107256. 38569937 PMC11063908

[48] Lee, M.‐J. 2018. “Transforming Growth Factor Beta Superfamily Regulation of Adipose Tissue Biology in Obesity.” Biochimica et Biophysica Acta, Molecular Basis of Disease 1864(4 Pt A): 1160–1171. 10.1016/j.bbadis.2018.01.025. 29409985

[49] Zhao, J. , L. Hu , W. Gui , L. Xiao , W. Wang , J. Xia , H. Fan , et al. 2022. “Hepatocyte TGF‐β Signaling Inhibiting WAT Browning to Promote NAFLD and Obesity Is Associated with Let‐7b‐5p.” Hepatol Commun 6(6): 1301–1321. 10.1002/hep4.1892. 35018737 PMC9134819

[50] Halbgebauer, D. , J. Roos , J. B. Funcke , H. Neubauer , B. S. Hamilton , E. Simon , E. Z. Amri , et al. 2021. “Latent TGFβ‐Binding Proteins Regulate UCP1 Expression and Function via TGFβ2.” Molecular Metabolism 53: 101336. 10.1016/j.molmet.2021.101336. 34481123 PMC8456047

[51] Woo, J. , C. Koziol‐White , R. Panettieri , and J. Jude . 2021. “TGF‐Β: The Missing Link in Obesity‐Associated Airway Diseases?” Curr Res Pharmacol Drug Discov 2: 100016. 10.1016/j.crphar.2021.100016. 34909651 PMC8663968

[52] Patel, S. , A. Alvarez‐Guaita , A. Melvin , D. Rimmington , A. Dattilo , E. L. Miedzybrodzka , I. Cimino , et al. 2019. “GDF15 Provides an Endocrine Signal of Nutritional Stress in Mice and Humans.” Cell Metabolism 29(3): 707–718.e8. 10.1016/j.cmet.2018.12.016. 30639358 PMC6408327

[53] Chandrasekaran, P. , and R. Weiskirchen . 2024. “The Role of Obesity in Type 2 Diabetes Mellitus—An Overview.” International Journal of Molecular Sciences 25(3): 1882. 10.3390/ijms25031882. 38339160 PMC10855901

[54] Liu, H. , and Y.‐G. Chen . 2022. “The Interplay between TGF‐β Signaling and Cell Metabolism.” Frontiers in Cell and Developmental Biology 10: 846723. 10.3389/fcell.2022.846723. 35359452 PMC8961331

[55] Liu, Y.‐Y. , D. Huo , L.‐T. Zeng , G.‐Q. Fan , T. Shen , T.‐M. Zhang , J.‐P. Cai , and J. Cui . 2022. “Mesencephalic Astrocyte‐Derived Neurotrophic Factor (MANF): Structure, Functions and Therapeutic Potential.” Ageing Research Reviews 82: 101763. 10.1016/j.arr.2022.101763. 36272696

[56] Schmitz, M. , M. Shaban , B. Albert , A. Gökçen , and M. Kracht . 2018. “The Crosstalk of Endoplasmic Reticulum (ER) Stress Pathways with NF‐Κb: Complex Mechanisms Relevant for Cancer, Inflammation and Infection.” Biomedicines 6(2): 58. 10.3390/biomedicines6020058. 29772680 PMC6027367

[57] Wu, S. A. , S. Kersten , and L. Qi . 2021. “Lipoprotein Lipase and its Regulators: An Unfolding Story.” Trends in Endocrinology and Metabolism 32(1): 48–61. 10.1016/j.tem.2020.11.005. 33277156 PMC8627828

[58] Junjappa, R. P. , P. Patil , K. R. Bhattarai , H.‐R. Kim , and H.‐J. Chae . 2018. “IRE1α Implications in Endoplasmic Reticulum Stress‐Mediated Development and Pathogenesis of Autoimmune Diseases.” Frontiers in Immunology 9: 1289. 10.3389/fimmu.2018.0128. 29928282 PMC5997832

[59] Shariq, O. A. , and T. J. McKenzie . 2020. “Obesity‐related Hypertension: a Review of Pathophysiology, Management, and the Role of Metabolic Surgery.” Gland Surgery 9(1): 80–93. 10.21037/gs.2019.12.03. 32206601 PMC7082272

[60] Flack, J. M. , D. Calhoun , and E. L. Schiffrin . 2017. “The New ACC/AHA Hypertension Guidelines for the Prevention, Detection, Evaluation, and Management of High Blood Pressure in Adults.” American Journal of Hypertension 31(2): 133–135. 10.1093/ajh/hpx207. 29228096

[61] Grundy, Scott M. 2016. “Metabolic Syndrome Update.” Trends in Cardiovascular Medicine 26(4): 364–373. 10.1016/j.tcm.2015.10.004. 26654259

[62] Lambert, E. A. , M. D. Esler , M. P. Schlaich , J. Dixon , N. Eikelis , and G. W. Lambert . 2019. “Obesity‐associated Organ Damage and Sympathetic Nervous Activity.” Hypertension 73(6): 1150–1159. 10.1161/HYPERTENSIONAHA.118.11676. 31067200

[63] Saxton, S. N. , B. J. Clark , S. B. Withers , E. C. Eringa , and A. M. Heagerty . 2019. “Mechanistic Links between Obesity, Diabetes, and Blood Pressure: Role of Perivascular Adipose Tissue.” Physiological Reviews 99(4): 1701–1763. 10.1152/physrev.00034.2018. 31339053

[64] Schütten, M. T. J. , A. J. H. M. Houben , P. W. de Leeuw , and C. D. A. Stehouwer . 2017. “The Link between Adipose Tissue Renin‐Angiotensin‐Aldosterone System Signaling and Obesity‐Associated Hypertension.” Physiology 32(3): 197–209. 10.1152/physiol.00037.2016. 28404736

[65] Cabandugama, P. K. , M. J. Gardner , and J. R. Sowers . 2017. “The Renin Angiotensin Aldosterone System in Obesity and Hypertension: Roles in the Cardiorenal Metabolic Syndrome.” Medical Clinics of North America 101(1): 129–137. 10.1016/j.mcna.2016.08.009. 27884224 PMC5125542

[66] Hall, J. E. , J. M. do Carmo , A. A. da Silva , Z. Wang , and M. E. Hall . 2019. “Obesity, Kidney Dysfunction and Hypertension: Mechanistic Links.” Nature Reviews Nephrology 15(6): 367–385. 10.1038/s41581-019-0145-4. 31015582 PMC7278043

[67] Bell, B. B. , and K. Rahmouni . 2016. “Leptin as a Mediator of Obesity‐Induced Hypertension.” Curr Obes Rep 5(4): 397–404. 10.1007/s13679-016-0231-x. 27665107 PMC5119542

[68] Shams, E. , V. Kamalumpundi , J. Peterson , R. A. Gismondi , W. Oigman , and M. L. de Gusmão Correia . 2022. “Highlights of Mechanisms and Treatment of Obesity‐Related Hypertension.” Journal of Human Hypertension 36(9): 785–793. 10.1038/s41371-021-00644-y. 35001082

[69] Chandrasekaran, P. , and R. Weiskirchen . 2024. “The Role of SCAP/SREBP as Central Regulators of Lipid Metabolism in Hepatic Steatosis.” International Journal of Molecular Sciences 25(2): 1109. 10.3390/ijms25021109. 38256181 PMC10815951

[70] Vekic, J. , A. Stefanovic , and A. Zeljkovic . 2023. “Obesity and Dyslipidemia: A Review of Current Evidence.” Curr Obes Rep 12(3): 207–222. 10.1007/s13679-023-00518-z. 37410248

[71] Vekic, J. , A. Zeljkovic , A. Stefanovic , Z. Jelic‐Ivanovic , and V. Spasojevic‐Kalimanovska . 2019. “Obesity and Dyslipidemia.” Metabolism 92: 71–81. 10.1016/j.metabol.2018.11.005. 30447223

[72] Chandrasekaran, P. , and R. Weiskirchen . 2024. “The Pivotal Role of the Membrane‐Bound O‐Acyltransferase Domain Containing 7 in Non‐alcoholic Fatty Liver Disease.” Liver 4(1): 1–14. 10.3390/livers4010001.

[73] Packard, C. J. , J. Boren , and M.‐R. Taskinen . 2020. “Causes and Consequences of Hypertriglyceridemia.” Frontiers in Endocrinology 11: 252. 10.3389/fendo.2020.00252. 32477261 PMC7239992

[74] Ravaut, G. , A. Légiot , K.‐F. Bergeron , and C. Mounier . 2021. “Monounsaturated Fatty Acids in Obesity‐Related Inflammation.” International Journal of Molecular Sciences 22(1): 330. 10.3390/ijms22010330. PMC7795523 33396940

[75] Superko, H. , and B. Garrett . 2022. “Small Dense LDL: Scientific Background, Clinical Relevance, and Recent Evidence Still a Risk Even with 'normal' LDL‐C Levels.” Biomedicines 10(4): 829. 10.3390/biomedicines10040829. 35453579 PMC9025822

[76] Björnson, E. , M. Adiels , M.‐R. Taskinen , and Jan Borén . 2017. “Kinetics of Plasma Triglycerides in Abdominal Obesity.” Current Opinion in Lipidology 28(1): 11–18. 10.1097/MOL.0000000000000375. 27898581

[77] Ference, B. A. , J. J. P. Kastelein , and A. L. Catapano . 2020. “Lipids and Lipoproteins in 2020.” JAMA 324(6): 595–596. 10.1001/jama.2020.5685. 32717042

[78] Yanai, H. , and H. Yoshida . 2019. “Beneficial Effects of Adiponectin on Glucose and Lipid Metabolism and Atherosclerotic Progression: Mechanisms and Perspectives.” International Journal of Molecular Sciences 20(5): 1190. 10.3390/ijms20051190. 30857216 PMC6429491

[79] Morofuji, Y. , S. Nakagawa , K. Ujifuku , T. Fujimoto , K. Otsuka , M. Niwa , and K. Tsutsumi . 2022. “Beyond Lipid‐Lowering: Effects of Statins on Cardiovascular and Cerebrovascular Diseases and Cancer.” Pharmaceuticals 15(2): 151. 10.3390/ph15020151. 35215263 PMC8877351

[80] Wolfe, B. M. , E. Kvach , and R. H. Eckel . 2016. “Treatment of Obesity: Weight Loss and Bariatric Surgery.” Circulation Research 118(11): 1844–1855. 10.1161/CIRCRESAHA.116.307591. 27230645 PMC4888907

[81] Polyzos, S. A. , J. Kountouras , and C. S. Mantzoros . 2019. “Obesity and Nonalcoholic Fatty Liver Disease: From Pathophysiology to Therapeutics.” Metabolism 92: 82–97. 10.1016/j.metabol.2018.11.014. 30502373

[82] Ress, C. , and S. Kaser . 2016. “Mechanisms of Intrahepatic Triglyceride Accumulation.” World Journal of Gastroenterology 22(4): 1664–1673. 10.3748/wjg.v22.i4.1664. 26819531 PMC4721997

[83] Rinella, M. E. , J. V. Lazarus , V. Ratziu , S. M. Francque , A. J. Sanyal , F. Kanwal , D. Romero , et al. 2023. “A Multisociety Delphi Consensus Statement on New Fatty Liver Disease Nomenclature.” Hepatology 78(6): 1966–1986. 10.1097/HEP.0000000000000520. 37363821 PMC10653297

[84] Wu, T. , Q. Liu , Y. Li , H. Li , L. Chen , X. Yang , Q. Tang , et al. 2021. “Feeding‐induced Hepatokine, Manf, Ameliorates Diet‐Induced Obesity by Promoting Adipose Browning via P38 MAPK Pathway.” Journal of Experimental Medicine 218(6): e20201203. 10.1084/jem.20201203. 33856409 PMC8054200

[85] Smith, G. I. , D. C. Polidori , M. Yoshino , M. L. Kearney , B. W. Patterson , B. Mittendorfer , and S. Klein . 2020. “Influence of Adiposity, Insulin Resistance, and Intrahepatic Triglyceride Content on Insulin Kinetics.” Journal of Clinical Investigation 130(6): 3305–3314. 10.1172/JCI136756. 32191646 PMC7260030

[86] Samovski, D. , P. Dhule , T. Pietka , M. Jacome‐Sosa , E. Penrose , N.‐H. Son , C. R. Flynn , et al. 2018. “Regulation of Insulin Receptor Pathway and Glucose Metabolism by CD36 Signaling.” Diabetes 67(7): 1272–1284. 10.2337/db17-1226. 29748289 PMC6014550

[87] Song, Z. , A. Xiaoli , and F. Yang . 2018. “Regulation and Metabolic Ssgnificance of De Novo Lipogenesis in Adipose Tissues.” Nutrients 10(10): 1383. 10.3390/nu10101383. 30274245 PMC6213738

[88] Zhu, Z. , X. Zhang , Q. Pan , L. Zhang , and J. Chai . 2023. “In‐depth Analysis of De Novo Lipogenesis in Non‐alcoholic Fatty Liver Disease: Mechanism and Pharmacological Interventions.” Liver Res 7(4): 285–295. 10.1016/j.livres.2023.11.003. PMC11791917 39958779

[89] Prasun, P. , I. Ginevic , and K. Oishi . 2021. “Mitochondrial Dysfunction in Nonalcoholic Fatty Liver Disease and Alcohol Related Liver Disease.” Transl Gastroenterol Hepatol 6: 4. 10.21037/tgh-20-125. 33437892 PMC7792990

[90] Emerenziani, S. , M. Pier Luca Guarino , L. Trillo Asensio , A. Altomare , M. Ribolsi , P. Balestrieri , and M. Cicala . 2019. “Role of Overweight and Obesity in Gastrointestinal Disease.” Nutrients 12(1): 111. 10.3390/nu12010111. 31906216 PMC7019431

[91] Karczewski, J. , B. Begier‐Krasińska , R. Staszewski , E. Popławska , K. Gulczynska‐Elhadi , and A. Dobrowolska . 2019. “Obesity and the Risk of Gastrointestinal Cancers.” Digestive Diseases and Sciences 64(10): 2740–2749. 10.1007/s10620-019-05603-9. 30968228 PMC6744518

[92] O'Sullivan, J. , J. Lysaght , C. L. Donohoe , and J. V. Reynolds . 2018. “Obesity and Gastrointestinal Cancer: the Interrelationship of Adipose and Tumour Microenvironments.” Nature Reviews Gastroenterology & Hepatology 15(11): 699–714. 10.1038/s41575-018-0069-7. 30323319

[93] Thalheimer, A. , and M. Bueter . 2021. “Excess Body Weight and Gastroesophageal Reflux Disease.” Visceral Medicine 37(4): 267–272. 10.1159/000516050. 34540942 PMC8406336

[94] Paris, S. , R. Ekeanyanwu , Y. Jiang , D. Davis , S. J. Spechler , and R. F. Souza . 2021. “Obesity and its Effects on the Esophageal Mucosal Barrier.” American Journal of Physiology ‐ Gastrointestinal and Liver Physiology 321(3): G335–G343. 10.1152/ajpgi.00199.2021. 34405732

[95] Miron, I. , and D. L. Dumitrascu . 2019. “Gastrointestinal Motility Disorders in Obesity.” Acta Endocrinologica 15(4): 497–504. 10.4183/aeb.2019.497. 32377248 PMC7200119

[96] Aune, D. , T. Norat , and L. J. Vatten . 2015. “Body Mass Index, Abdominal Fatness and the Risk of Gallbladder Disease.” European Journal of Epidemiology 30(9): 1009–1019. 10.1007/s10654-015-0081-y. 26374741

[97] Camilleri, M. , H. Malhi , and A. Acosta . 2017. “Gastrointestinal Complications of Obesity.” Gastroenterology 152(7): 1656–1670. 10.1053/j.gastro.2016.12.052. 28192107 PMC5609829

[98] Bonsignore, M. R. , P. Baiamonte , E. Mazzuca , A. Castrogiovanni , and O. Marrone . 2019. “Obstructive Sleep Apnea and Comorbidities: a Dangerous Liaison.” Multidiscip Respir Med 14: 8. 10.4081/mrm.2019.10. 30809382 PMC6374907

[99] Dong, Z. , X. Xu , C. Wang , S. Cartledge , R. Maddison , and S. M. Shariful Islam . 2020. “Association of Overweight and Obesity with Obstructive Sleep Apnoea: A Systematic Review and Meta‐Analysis.” Obesity Med 17: 100185. 10.1016/j.obmed.2020.100185.

[100] Kuvat, N. , H. Tanriverdi , and F. Armutcu . 2020. “The Relationship between Obstructive Sleep Apnea Syndrome and Obesity: A New Perspective on the Pathogenesis in Terms of Organ Crosstalk.” The Clinical Researcher J 14(7): 595–604. 10.1111/crj.13175. 32112481

[101] Baron, K. G. , K. J. Reid , T. Kim , L. Van Horn , H. Attarian , L. Wolfe , J. Siddique , G. Santostasi , and P. C. Zee . 2017. “Circadian Timing and Alignment in Healthy Adults: Associations with BMI, Body Fat, Caloric Intake and Physical Activity.” International Journal of Obesity 41(2): 203–209. 10.1038/ijo.2016.194. 27795550 PMC5296236

[102] Jehan, S. , E. Auguste , F. Zizi , S. R. Pandi‐Perumal , R. Gupta , H. Attarian , G. Jean‐Louis , and S. I. McFarlane . 2016. “Obstructive Sleep Apnea: Women's Perspective.” J Sleep Med Disord 3(6): 1064: PMID: 28239685. 28239685 PMC5323064

[103] Barber, T. M. , P. Hanson , M. O. Weickert , and S. Franks . 2019. “Obesity and Polycystic Ovary Syndrome: Implications for Pathogenesis and Novel Management Strategies.” Clinical Medicine Insights: Reproductive Health 13: 1179558119874042. 10.1177/1179558119874042. 31523137 PMC6734597

[104] Silvestris, E. , G. de Pergola , R. Rosania , and G. Loverro . 2018. “Obesity as Disruptor of the Female Fertility.” Reproductive Biology and Endocrinology 16(1): 22. 10.1186/s12958-018-0336-z. 29523133 PMC5845358

[105] Barber, T. M. , and S. Franks . 2021. “Obesity and Polycystic Ovary Syndrome.” Clinical Endocrinology 95(4): 531–541. 10.1111/cen.14421. 33460482

[106] Zhao, H. , J. Zhang , X. Cheng , X. Nie , and B. He . 2023. “Insulin Resistance in Polycystic Ovary Syndrome across Various Tissues: an Updated Review of Pathogenesis, Evaluation, and Treatment.” Journal of Ovarian Research 16(1): 9. 10.1186/s13048-022-01091-0. 36631836 PMC9832677

[107] Ollila, M.‐M. E. , T. Piltonen , K. Puukka , A. Ruokonen , M.‐R. Järvelin , J. S. Tapanainen , S. Franks , and L. Morin‐Papunen . 2016. “Weight Gain and Dyslipidemia in Early Adulthood Associate with Polycystic Ovary Syndrome: Prospective Cohort Study.” Journal of Clinical Endocrinology & Metabolism 101(2): 739–747. 10.1210/jc.2015-3543. 26652764

[108] Ek, I. , P. Arner , M. Rydén , C. Holm , A. Thörne , J. Hoffstedt , and H. Wahrenberg . 2002. “A Unique Defect in the Regulation of Visceral Fat Cell Lipolysis in the Polycystic Ovary Syndrome as an Early Link to Insulin Resistance.” Diabetes 51(2): 484–492. 10.2337/diabetes.51.2.484. 11812759

[109] O'Reilly, M. W. , P. J. House , and J. W. Tomlinson . 2014. “Understanding Androgen Action in Adipose Tissue.” The Journal of Steroid Biochemistry and Molecular Biology 143: 277–284. 10.1016/j.jsbmb.2014.04.008. 24787657

[110] Öztürk, A. , S. K. Kucur , A. Seven , E. Deveci , H. Şencan , O. Yilmaz , and A. Kiliç . 2019. “Temperament and Character Differences of Patients with Polycystic Ovary Syndrome.” J Gynecol Obstet Hum Reprod 48(4): 255–259. 10.1016/j.jogoh.2019.01.006. 30711689

[111] Karjula, S. , L. Morin‐Papunen , J. Auvinen , A. Ruokonen , K. Puukka , S. Franks , M.‐R. Järvelin , et al. 2017. “Psychological Distress Is More Prevalent in Fertile Age and Premenopausal Women with PCOS Symptoms: 15‐year Follow‐Up.” Journal of Clinical Endocrinology & Metabolism 102(6): 1861–1869. 10.1210/jc.2016-3863. 28323926 PMC5470769

[112] Wondmkun, Y. T. 2020. “Obesity, Insulin Resistance, and Type 2 Diabetes: Associations and Therapeutic Implications.” Diabetes Metab Syndr Obes 13: 3611–3616. 10.2147/DMSO.S275898. 33116712 PMC7553667

[113] Smith, G. I. , M. Shankaran , M. Yoshino , G. G. Schweitzer , M. Chondronikola , J. W. Beals , A. L. Okunade , et al. 2020. “Insulin Resistance Drives Hepatic De Novo Lipogenesis in Nonalcoholic Fatty Liver Disease.” Journal of Clinical Investigation 130(3): 1453–1460. 10.1172/JCI134165. 31805015 PMC7269561

[114] Crewe, C. , Y. A. An , and P. E. Scherer . 2017. “The Ominous Triad of Adipose Tissue Dysfunction: Inflammation, Fibrosis, and Impaired Angiogenesis.” Journal of Clinical Investigation 127(1): 74–82. 10.1172/JCI88883. 28045400 PMC5199684

[115] Seo, J. B. , M. Riopel , P. Cabrales , J. Y. Huh , G. K. Bandyopadhyay , A. Y. Andreyev , A. N. Murphy , et al. 2019. “Knockdown of Ant2 Reduces Adipocyte Hypoxia and Improves Insulin Resistance in Obesity.” Nature Metabolism 1(1): 86–97. 10.1038/s42255-018-0003-x. PMC6746433 31528845

[116] Straub, L. G. , and P. E. Scherer . 2019. “Metabolic Messengers: Adiponectin.” Nature Metabolism 1(3): 334–339. 10.1038/s42255-019-0041-z. PMC7357716 32661510

[117] Petersen, M. C. , and G. I. Shulman . 2018. “Mechanisms of Insulin Action and Insulin Resistance.” Physiological Reviews 98(4): 2133–2223. 10.1152/physrev.00063.2017. 30067154 PMC6170977

[118] Cifarelli, V. , S. C. Beeman , G. I. Smith , J. Yoshino , D. Morozov , J. W. Beals , B. D. Kayser , et al. 2020. “Decreased Adipose Tissue Oxygenation Associates with Insulin Resistance in Individuals with Obesity.” Journal of Clinical Investigation 130(12): 6688–6699. 10.1172/JCI141828. 33164985 PMC7685757

[119] Lewis, G. F. , A. C. Carpentier , S. Pereira , M. Hahn , and A. Giacca . 2021. “Direct and Indirect Control of Hepatic Glucose Production by Insulin.” Cell Metabolism 33(4): 709–720. 10.1016/j.cmet.2021.03.007. 33765416

[120] Wu, H. , and C. M. Ballantyne . 2020. “Metabolic Inflammation and Insulin Resistance in Obesity.” Circulation Research 126(11): 1549–1564. 10.1161/CIRCRESAHA.119.315896. 32437299 PMC7250139

[121] Wang, X. , M. Xu , and Y. Li . 2022. “Adipose Tissue Aging and Metabolic Disorder, and the Impact of Nutritional Interventions.” Nutrients 14(15): 3134. 10.3390/nu14153134. 35956309 PMC9370499

[122] Zhang, J. , J.‐H. Xu , Q.‐Q. Qu , and G.‐Q. Zhong . 2020. “Risk of Cardiovascular and Cerebrovascular Events in Polycystic Ovarian Syndrome Women: A Meta‐Analysis of Cohort Studies.” Front Cardiovasc Med 7: 552421. 10.3389/fcvm.2020.552421. 33282917 PMC7690560

[123] Yao, J. , D. Wu , and Y. Qiu . 2022. “Adipose Tissue Macrophage in Obesity‐Associated Metabolic Diseases.” Frontiers in Immunology 13: 977485. 10.3389/fimmu.2022.977485. 36119080 PMC9478335

[124] Csige, I. , D. Ujvárosy , Z. Szabó , I. Lőrincz , G. Paragh , M. Harangi , and S. Somodi . 2018. “The Impact of Obesity on the Cardiovascular System.” Journal of Diabetes Research 2018: 3407306–3407312. 10.1155/2018/3407306. 30525052 PMC6247580

[125] Jankowski, Joachim , Jürgen Floege , Danilo Fliser , Michael Böhm , and Nikolaus Marx . 2021. “Cardiovascular Disease in Chronic Kidney Disease.” Circulation 143(11): 1157–1172. 10.1161/CIRCULATIONAHA.120.050686. 33720773 PMC7969169

[126] Sewell, J. , S. M. Hussain , Y. Wang , A. E. Wluka , Y. Z. Lim , M. J. Carrington , K. Samaras , and F. M. Cicuttini . 2022. “Association between Arthritis and Cardiovascular Risk Factors in Community‐Based Adults: an Opportunity to Target Cardiovascular Risk.” BMC Cardiovascular Disorders 22(1): 232. 10.1186/s12872-022-02674-x. 35590252 PMC9118727

[127] Alpert, M. A. , J. Omran , and B. P. Bostick . 2016. “Effects of Obesity on Cardiovascular Hemodynamics, Cardiac Morphology, and Ventricular Function.” Curr Obes Rep 5(4): 424–434. 10.1007/s13679-016-0235-6. 27744513

[128] Tietjens, J. R. , D. Claman , E. J. Kezirian , T. De Marco , A. Mirzayan , B. Sadroonri , A. N. Goldberg , C. Long , E. P. Gerstenfeld , and Y. Yeghiazarians . 2019. “Obstructive Sleep Apnea in Cardiovascular Disease: A Review of the Literature and Proposed Multidisciplinary Clinical Management Strategy.” Journal of the American Heart Association 8(1): e010440. 10.1161/JAHA.118.010440. 30590966 PMC6405725

[129] Shah, S. , A. Henry , C. Roselli , H. Lin , G. Sveinbjörnsson , G. Fatemifar , Å. K. Hedman , et al. 2020. “Genome‐wide Association and Mendelian Randomisation Analysis Provide Insights into the Pathogenesis of Heart Failure.” Nature Communications 11(1): 163. 10.1038/s41467-019-13690-5. PMC6952380 31919418

[130] Gruzdeva, O. , D. Borodkina , E. Uchasova , Y. Dyleva , and O. Barbarash . 2018. “Localization of Fat Depots and Cardiovascular Risk.” Lipids in Health and Disease 17(1): 218. 10.1186/s12944-018-0856-8. 30219068 PMC6138918

[131] Kajikawa, M. , and Y. Higashi . 2022. “Obesity and Endothelial Function.” Biomedicines 10(7): 1745. 10.3390/biomedicines10071745. 35885049 PMC9313026

[132] Keane, K. N. , V. F. Cruzat , R. Carlessi , P. I. H. de Bittencourt , and P. Newsholme . 2015. “Molecular Events Linking Oxidative Stress and Inflammation to Insulin Resistance and β‐cell Dysfunction.” Oxidative Medicine and Cellular Longevity 2015: 181643–181715. 10.1155/2015/181643. 26257839 PMC4516838

[133] Manoharan, M. P. , R. Raja , A. Jamil , D. Csendes , S. D. Gutlapalli , K. Prakash , K. M. Swarnakari , et al. 2022. “Obesity and Coronary Artery Disease: An Updated Systematic Review 2022.” Cureus 14(9): e29480. 10.7759/cureus.29480. 36299943 PMC9588166

[134] Lopez‐Jimenez, F. , W. Almahmeed , H. Bays , A. Cuevas , E. Di Angelantonio , C. W. le Roux , N. Sattar , et al. 2022. “Obesity and Cardiovascular Disease: Mechanistic Insights and Management Strategies. A Joint Position Paper by the World Heart Federation and World Obesity Federation.” Eur J Prev Cardiol 29(17): 2218–2237. 10.1093/eurjpc/zwac187. 36007112

[135] Pati, S. , W. Irfan , A. Jameel , S. Ahmed , and R. K. Shahid . 2023. “Obesity and Cancer: A Current Overview of Epidemiology, Pathogenesis, Outcomes, and Management.” Cancers 15(2): 485. 10.3390/cancers15020485. 36672434 PMC9857053

[136] Sánchez‐Jiménez, F. , A. Pérez‐Pérez , L. de la Cruz‐Merino , and V. Sánchez‐Margalet . 2019. “Obesity and Breast Cancer: Role of Leptin.” Frontiers Oncology 9: 596. 10.3389/fonc.2019.00596. PMC6657346 31380268

[137] Tumminia, A. , F. Vinciguerra , M. Parisi , M. Graziano , L. Sciacca , R. Baratta , and L. Frittitta . 2019. “Adipose Tissue, Obesity and Adiponectin: Role in Endocrine Cancer Risk.” International Journal of Molecular Sciences 20(12): 2863. 10.3390/ijms20122863. 31212761 PMC6628240

[138] Gui, Y. , Q. Pan , X. Chen , S. Xu , X. Luo , and L. Chen . 2017. “The Association between Obesity Related Adipokines and Risk of Breast Cancer: a Meta‐Analysis.” Oncotarget 8(43): 75389–75399. 10.18632/oncotarget.17853. 29088874 PMC5650429

[139] Perry, R. J. , and G. I. Shulman . 2020. “Mechanistic Links between Obesity, Insulin, and Cancer.” Trends Cancer 6(2): 75–78. 10.1016/j.trecan.2019.12.003. 32061306 PMC7214048

[140] Wishart, A. L. , S. J. Conner , J. R. Guarin , J. P. Fatherree , Y. Peng , R. A. McGinn , R. Crews , et al. 2020. “Decellularized Extracellular Matrix Scaffolds Identify Full‐Length Collagen VI as a Driver of Breast Cancer Cell Invasion in Obesity and Metastasis.” Science Advances 6(43): eabc3175. 10.1126/sciadv.abc3175. 33087348 PMC7577726

[141] Druso, J. E. , and C. Fischbach . 2018. “Biophysical Properties of Extracellular Matrix: Linking Obesity and Cancer.” Trends Cancer 4(4): 271–273. 10.1016/j.trecan.2018.02.001. 29606310 PMC5884448

[142] Hao, J. , Y. Zhang , X. Yan , F. Yan , Y. Sun , J. Zeng , S. Waigel , et al. 2018. “Circulating Adipose Fatty Acid Binding Protein Is a New Link Underlying Obesity‐Associated Breast/mammary Tumor Development.” Cell Metabolism 28(5): 689–705.e5. 10.1016/j.cmet.2018.07.006. 30100196 PMC6221972

[143] Ringel, A. E. , J. M. Drijvers , G. J. Baker , A. Catozzi , J. C. García‐Cañaveras , B. M. Gassaway , B. C. Miller , et al. 2020. “Obesity Shapes Metabolism in the Tumor Microenvironment to Suppress Anti‐tumor Immunity.” Cell 183(7): 1848–1866.e26. 10.1016/j.cell.2020.11.009. 33301708 PMC8064125

[144] Tiwari, P. , A. Blank , C. Cui , K. Q. Schoenfelt , G. Zhou , Y. Xu , G. Khramtsova , et al. 2019. “Metabolically Activated Adipose Tissue Macrophages Link Obesity to Triple‐Negative Breast Cancer.” Journal of Experimental Medicine 216(6): 1345–1358. 10.1084/jem.20181616. 31053611 PMC6547867

[145] Popoviciu, M.‐S. , L. Păduraru , G. Yahya , K. Metwally , and S. Cavalu . 2023. “Emerging Role of GLP‐1 Agonists in Obesity: A Comprehensive Review of Randomised Controlled Trials.” International Journal of Molecular Sciences 24(13): 10449. 10.3390/ijms241310449. 37445623 PMC10341852

[146] Cheang, J. Y. , and P. M. Moyle . 2018. “Glucagon‐like Peptide‐1 (GLP‐1)‐Based Therapeutics: Current Status and Future Opportunities beyond Type 2 Diabetes.” ChemMedChem 13(7): 662–671. 10.1002/cmdc.201700781. 29430842

[147] Wang, L. 2022. “Designing a Dual GLP‐1R/GIPR Agonist from Tirzepatide: Comparing Residues between Tirzepatide, GLP‐1, and GIP.” Drug Design, Development and Therapy 16: 1547–1559. 10.2147/dddt.S358989. 35651477 PMC9149770

[148] Rubino, D. , N. Abrahamsson , M. Davies , D. Hesse , F. L. Greenway , C. Jensen , I. Lingvay , et al. 2021. “Effect of Continued Weekly Subcutaneous Semaglutide vs Placebo on Weight Loss Maintenance in Adults with Overweight or Obesity: The STEP 4 Randomized Clinical Trial.” JAMA 325(14): 1414–1425. 10.1001/jama.2021.3224. 33755728 PMC7988425

[149] Harada, M. 2022. “Pathophysiology of Polycystic Ovary Syndrome Revisited: Current Understanding and Perspectives Regarding Future Research.” Reproductive Medicine and Biology 21(1): e12487. 10.1002/rmb2.12487. 36310656 PMC9601867

[150] Wang, D. , N. Nan , H. Bing , and B. He . 2023. “Controlled Attenuation Parameters to Assess Liver Steatosis in Obese Patients with Polycystic Ovary Syndrome.” Frontiers in Endocrinology 14: 1241734. 10.3389/fendo.2023.1241734. 37720537 PMC10501797

[151] Zinman, B. , M. A. Nauck , H. Bosch‐Traberg , H. Frimer‐Larsen , D. D. Ørsted , and J. B. Buse . 2018. “Liraglutide and Glycaemic Outcomes in the LEADER Trial.” Diabetes Ther 9(6): 2383–2392. 10.1007/s13300-018-0524-z. 30392095 PMC6250637

[152] Gu, J. , X. Meng , Y. Guo , L. Wang , H. Zheng , Y. Liu , B. Wu , and D. Wang . 2016. “The Efficacy and Safety of Liraglutide Added to Metformin in Patients with Diabetes: a Meta‐Analysis of Randomized Controlled Trials.” Scientific Reports 6(1): 32714. 10.1038/srep32714. 27600499 PMC5013324

[153] Kadowaki, T. , J. Isendahl , U. Khalid , S. Y. Lee , T. Nishida , W. Ogawa , K. Tobe , T. Yamauchi , and S. Lim . , and STEP 6 investigators . 2022. “Semaglutide once a Week in Adults with Overweight or Obesity, with or without Type 2 Diabetes in an East Asian Population (STEP 6): a Randomised, Double‐Blind, Double‐Dummy, Placebo‐Controlled, Phase 3a Trial.” Lancet Diabetes & Endocrinology 10(3): 193–206. 10.1016/S2213-8587(22)00008-0. 35131037

[154] Rubino, D. M. , F. L. Greenway , U. Khalid , P. M. O'Neil , J. Rosenstock , R. Sørrig , T. A. Wadden , et al. 2022. “Effect of Weekly Subcutaneous Semaglutide vs Daily Liraglutide on Body Weight in Adults with Overweight or Obesity without Diabetes: The STEP 8 Randomized Clinical Trial.” JAMA 327(2): 138–150. 10.1001/jama.2021.23619. 35015037 PMC8753508

[155] Dahl, D. , Y. Onishi , P. Norwood , R. Huh , R. Bray , H. Patel , and Á. Rodríguez . 2022. “Effect of Subcutaneous Tirzepatide vs Placebo Added to Titrated Insulin Glargine on Glycemic Control in Patients with Type 2 Diabetes: The SURPASS‐5 Randomized Clinical Trial.” JAMA 327(6): 534–545. 10.1001/jama.2022.0078. 35133415 PMC8826179

[156] Jastreboff, A. M. , L. J. Aronne , N. N. Ahmad , S. Wharton , L. Connery , B. Alves , A. Kiyosue , et al. 2022. “Tirzepatide once Weekly for the Treatment of Obesity.” New England Journal of Medicine 387(3): 205–216. 10.1056/NEJMoa2206038. 35658024

